# Recombination suppression in heterozygotes for a pericentric inversion induces the interchromosomal effect on crossovers in Arabidopsis

**DOI:** 10.1111/tpj.14505

**Published:** 2019-10-07

**Authors:** Pasquale Termolino, Matthieu Falque, Riccardo Aiese Cigliano, Gaetana Cremona, Rosa Paparo, Antoine Ederveen, Olivier C. Martin, Federica M. Consiglio, Clara Conicella

**Affiliations:** ^1^ Institute of Biosciences and Bioresources (IBBR) National Research Council of Italy (CNR) 80055 Portici Italy; ^2^ Génétique Quantitative et Evolution‐Le Moulon Institut National de la Recherche Agronomique Université Paris‐Sud CNRS AgroParisTech Université Paris‐Saclay 91190 Gif‐sur‐Yvette France; ^3^ Sequentia Biotech Edifici Eureka, Campus UAB 08005 Barcelona Spain; ^4^ Department of Molecular Plant Physiology Institute for Water and Wetland Research (IWWR) Radboud University Nijmegen 9102 6500 Nijmegen the Netherlands

**Keywords:** meiosis, recombination, crossing over, crossover interference, chromosome rearrangement, interchromosomal effect

## Abstract

During meiosis, recombination ensures allelic exchanges through crossovers (COs) between the homologous chromosomes. Advances in our understanding of the rules of COs have come from studies of mutations including structural chromosomal rearrangements that, when heterozygous, are known to impair COs in various organisms. In this work, we investigate the effect of a large heterozygous pericentric inversion on male and female recombination in Arabidopsis. The inversion was discovered in the *Atmcc1* mutant background and was characterized through genetic and next‐generation sequencing analysis. Reciprocal backcross populations, each consisting of over 400 individuals, obtained from the mutant and the wild type, both crossed with Landsberg *erecta*, were analyzed genome‐wide by 143 single‐nucleotide polymorphisms. The negative impact of inversion became evident in terms of CO loss in the rearranged chromosome in both male and female meiosis. No single‐CO event was detected within the inversion, consistent with a post‐meiotic selection operating against unbalanced gametes. Cytological analysis of chiasmata in F_1_ plants confirmed that COs were reduced in male meiosis in the chromosome with inversion. Crossover suppression on the rearranged chromosome is associated with a significant increase of COs in the other chromosomes, thereby maintaining unchanged the number of COs per cell. The CO pattern observed in our study is consistent with the interchromosomal (IC) effect as first described in *Drosophila*. In contrast to male meiosis, in female meiosis no IC effect is visible. This may be related to the greater strength of interference that constrains the CO number in excess of the minimum value imposed by CO assurance in Arabidopsis female meiosis.

## Significance Statement

This work provides further information into the poorly described phenomenon of the interchromosomal (IC) effect in plants. Indeed, the few studies on this phenomenon in maize date back to the 1960s and 1970s and have low resolution, being limited to a few specific regions and genetic markers. In this paper we underscore the significance for plant scientists of the IC effect during meiosis and provide more detailed data and analyses.

## Introduction

Meiotic recombination is fundamental in the creation of genetic diversity through the shuffling of alleles over generations. Recombination is initiated by DNA double‐strand breaks (DSBs) that are partly repaired as crossovers (COs), i.e. through reciprocal exchanges between the homologous chromosomes. Since more DSBs are generally formed than COs, alternative pathways of DSB repair occur as non‐crossovers (NCOs), i.e. non reciprocal exchange and inter‐sister repair (IS) (Lambling *et al*., 2017). Previous studies suggest that the CO rate is a trait under selection for its lower as well as upper limits (Ritz *et al*., 2017). Since CO formation is subject to numerous constraints, to ensure proper chromosome segregation at meiosis I, at least one CO per homologous chromosome pair (obligatory CO) has to occur (Jones and Franklin, 2006), while the formation of excess COs is almost always limited by mechanical factors (such as entanglement) or evolutionary pressures (the putative mutagenicity of COs) (Arbeithuber *et al*., 2015). The complex CO control system includes CO homeostasis that maintains a nearly constant number of COs per meiosis despite variation in the number of DSBs (Martini *et al*., 2006) and CO interference that leads COs to be farther apart along the chromosome than expected by chance (Berchowitz and Copenhaver, 2010). The molecular mechanisms underlying CO homeostasis and interference are not well understood. Crossover homeostasis was first documented in yeast (Martini *et al*., 2006) and subsequently found in other organisms (Cole *et al*., 2012; Yokoo *et al*., 2012). In yeast and *Caenorhabditis elegans* a feedback loop mechanism is thought to impose the formation of additional DSBs until the number of DSBs is sufficient to obtain an adequate CO rate (Lao *et al*., 2013; Rosu *et al*., 2013; Thacker *et al*., 2014). In maize and Arabidopsis the extent of homeostasis is limited (Sidhu *et al*., 2015; Xue *et al*., 2018). In particular, in maize the number of DSBs is highly variable among inbred lines and is strongly correlated with CO number, which ranges from 11 to 19 (Sidhu *et al*., 2015). The majority of COs are subject to genetic interference, as reported in numerous species. For example, in Arabidopsis and maize about 85% of total COs are sensitive to interference (Falque *et al*., 2009; Drouaud *et al*., 2013). Mutations in components or pathways limiting COs allow for an increase in the number of COs typical of a given organism. For example, partial depletion of proteins of the central region of the synaptonemal complex increases COs in *C. elegans* (Libuda *et al*., 2013). In Arabidopsis the disruption of pathways that drive DSB repair towards NCOs induces an almost eight‐fold boost in the CO rate, apparently without any negative consequence for genome stability (Fernandes *et al*., 2018). On the other hand, the lower limit of the number of COs, referred to as ‘CO assurance’, should be maintained by the obligatory CO. In natural populations, mechanisms that decrease COs (but not below the lower limit) are probably tolerated because they maintain adaptive allele combinations which otherwise would be broken by recombination (Thompson and Jiggins, 2014). One of these mechanisms operates through inversions, which are strong suppressors of recombination across the rearranged chromosomal regions (Fransz *et al*., 2016). Suppression of COs in inverted regions is reported in various organisms including yeast (Dresser *et al*., 1994), *C. elegans* (Zetka and Rose, 1992), mammals (Massip *et al*., 2010; Kirkpatrick *et al*., 2012) and plants (Lamb *et al*., 2007; Ederveen *et al*., 2015; Zapata *et al*., 2016). Inversions have been studied extensively in multiple *Drosophila* species (Stevison *et al*., 2011). Although heterozygous inversions suppress COs in the rearranged region, they may enhance them on the remaining chromosomes in a phenomenon referred to as the ‘interchromosomal effect’, a nomenclature that will be used hereafter (for a review see Lucchesi and Suzuki, 1968). The interchromosomal (IC) effect, first described in *Drosophila*, has been found in grasshopper (White and Morley, 1955), maize (Bellini and Bianchi, 1963; McKinley and Goldman, 1979) and maize–teosinte hybrids (Ting, 1963).

In this work, we provide insights into the IC effect in Arabidopsis. A pericentric inversion occurring in chromosome 3 was ascertained in heterozygous populations of the *meiotic control of crossover 1* (*Atmcc1*) mutant and confirmed by next‐generation sequencing (NGS) in the homozygous line. We show that *Atmcc1*, along with T‐DNA mutation previously described by Perrella *et al*. (2010), carries a large inversion that is probably a side effect of T‐DNA integration since chromosomal rearrangements are common at T‐DNA sites (Kleinboelting *et al*., 2015). We examine the effect of the inversion on CO rate in both female and male meiosis. The impact of the inversion was a loss of COs in the rearranged chromosome 3 and, in male meiosis, a significant increase in COs in the other chromosomes, which is consistent with the IC effect.

## Results

### Recombination analysis of *Atmcc1* heterozygous populations reveals loss of COs on chromosome 3

**Figure 1:**
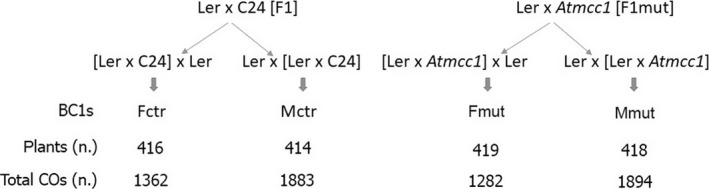
Scheme of the four reciprocal backcrosses (BC1s) obtained from *Atmcc1* mutant and wild type Arabidopsis plants including the number of BC1 plants and total crossovers (COs). Fctr, Mctr, BC1s from F_1_ wild‐type plants used as the female and male parent, respectively; Fmut, Mmut, BC1s from F_1_ mutant plants used as the female and male parent, respectively; L*er*, Landsberg *erecta* (recurrent parent), C24 = background of *Atmcc1*.

**Figure 2:**
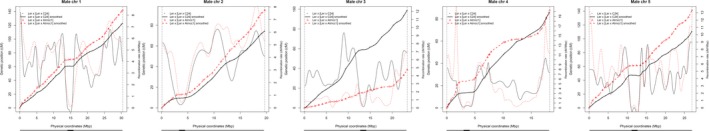
Relationship between physical (Mbp) and genetic positions (cM) of the markers on each chromosome, and the corresponding recombination rates (cM/Mbp) in male control (black) and male mutant (red) linkage maps. In the bar under each graph, the black box indicates the approximate centromere position according to the Arabidopsis TAIR (https://www.arabidopsis.org/).

We generated F_1_ progenies by crossing Landsberg *erecta* (L*er*) with homozygous *Atmcc1* (L*er* × *Atmcc*1) and used L*er* × C24 as the control. Subsequently, we crossed these F_1_ plants, as male and female, with L*er*, thereby obtaining four large reciprocal backcross (BC1) populations (Fctr, Mctr, Fmut, Mmut) (Figure 1). The number of COs, revealed by means of 143 single nucleotide polymorphisms (SNPs) analyzed in the four BC1 populations, were 1894 in Mmut (*n* = 418 plants) and 1282 in Fmut (*n* = 419 plants), while 1883 and 1362 COs were found in Mctr (*n* = 414 plants) and Fctr (*n* = 416 plants), respectively. The total number of COs is similar between the respective mut and ctr populations (Mmut versus Mctr; Fmut versus Fctr; *P* = 0.5, Fisher's test). Accordingly, the estimated average number of COs per cell is 9.06 versus 9.09 (Mmut versus Mctr) for male meiosis and 6.11 versus 6.55 (Fmut versus Fctr) for female meiosis. The number of COs is significantly different between male and female meiosis, in agreement with the known heterochiasmy (*P* = 0.02, chi‐square test). The relationship between the genetic map length in centimorgans and the physical length in megabase pairs was estimated from the Marey map for each chromosome (Figure 2 for male meiosis, Figure S1 in the online Supporting Information for female meiosis). The marker positions refer to L*er* as reported in the Weigel database in the ‘1001 Genomes’ project (Clark *et al*., 2007; Zeller *et al*., 2008). In control populations, the recombination rate in male (Figure 2) and female meiosis (Figure S1) is substantially in agreement with the results published by Giraut *et al*. (2011) even though the genotypes and markers used in our study are only partially the same. Male recombination tended to be higher towards the telomeres whereas pericentromeric regions were devoid of COs. As expected, the regions close to telomeres showed a lower recombination in female meiosis than male meiosis. In both mutant populations, the loss of COs in chromosome 3 was strikingly evident (Figures 2 and S1). Indeed, we found that the mean number of COs there was 0.54 versus 1.82 (Mmut versus Mctr) in male meiosis and 0.61 versus 1.12 (Fmut versus Fctr) in female meiosis. Additionally, we detected a gain of COs in Mmut versus Mctr for chromosomes 1, 2 and 5 (Figure 2). They exhibited average numbers of COs of 2.70 versus 2.34, 1.67 versus 1.34, and 2.63 versus 2.03, respectively.

### Chromosome 3 carries a large pericentric inversion

**Figure 3:**
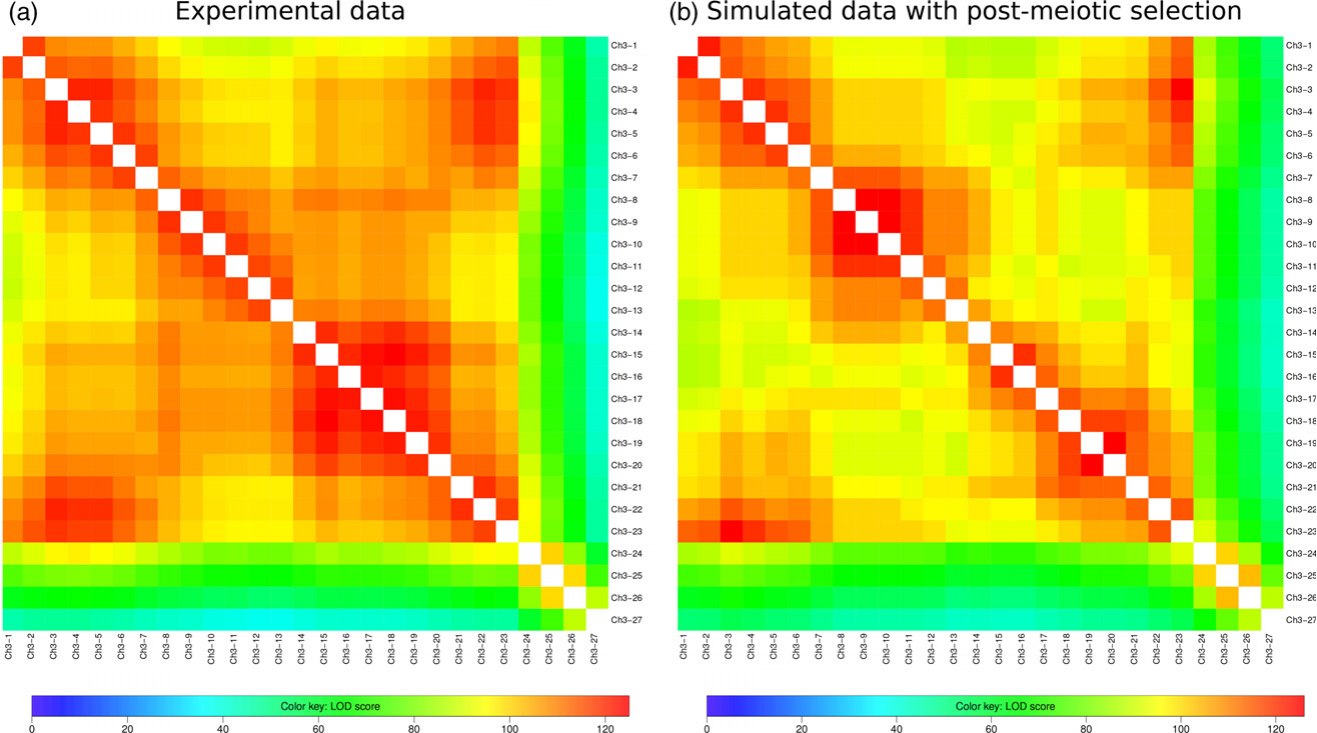
Heat maps for the two‐point linkage logarithm of the odds (LOD) scores on chromosome 3 with the inversion, in male meiosis. (a) Experimental data. (b) Prediction of the model simulating pairing, crossover formation and post‐meiotic selection. Note the characteristic block structure of these heat maps in which the boundaries of the inverted region are tightly linked (high LOD score).

To find an explanation for the reduction in COs in chromosome 3, we carefully examined the genetic map of this chromosome and also sequenced the *Atmcc1* genome. Twenty‐seven SNP markers of chromosome 3 (reported in Table S1) were used to construct a genetic map based on the maximum likelihood estimate. We found that the region extending from marker CH3‐3 to CH3‐23, which contains the centromere, differed in orientation of the markers between mutant and control. In addition, the pairwise two‐point linkage of chromosome 3 markers revealed a linkage much greater than expected between markers CH3‐3 and CH3‐23, resulting in a strong linkage of corresponding flanking markers in the mutant. Altogether, these findings point to a possible inversion of the segment spanning the CH3‐3 to CH3‐23 interval (Figures 3a and S2a). To confirm boundaries and identify break points in this putative pericentric inversion, a whole‐genome resequencing of *Atmcc1* was performed, generating paired‐end reads from a library of average fragment size equal to 267 bp. The analysis of the reads in regions flanked by markers CH3‐2/CH3‐3 and CH3‐23/CH3‐24 revealed 28 anomalous read pairs having the same orientation, of which the expected forward reads mapped to the position Chr3:20699839 and the expected reverse reads to the position Chr3:774808 (Figure S3). In addition, four split‐reads were found with a primary alignment at the position Chr3:20699839 and a secondary alignment at Chr3:774808, providing strong and direct evidence for the position of the inversion in *Atmcc1* (Figure S3). On the other hand, no reads with those characteristics were found in C24 resequencing data (Figure S4). Together, these results confirmed the presence of the pericentric inversion with the break points lying between the markers CH3‐2 and CH3‐3, and between CH3‐23 and CH3‐24 (Figure S5). This conclusion is exactly as predicted by the genetic mapping data. Relationships between physical and genetic maps on chromosome 3 (Figure 2) also showed that in the mutant recombination in the region between Chr3‐24 and Chr3‐27 was similar to that in the control, pointing to Chr3‐23 as the last marker within the inversion.

### Post‐meiotic selection occurs following recombination within the inversion

**Figure 4:**
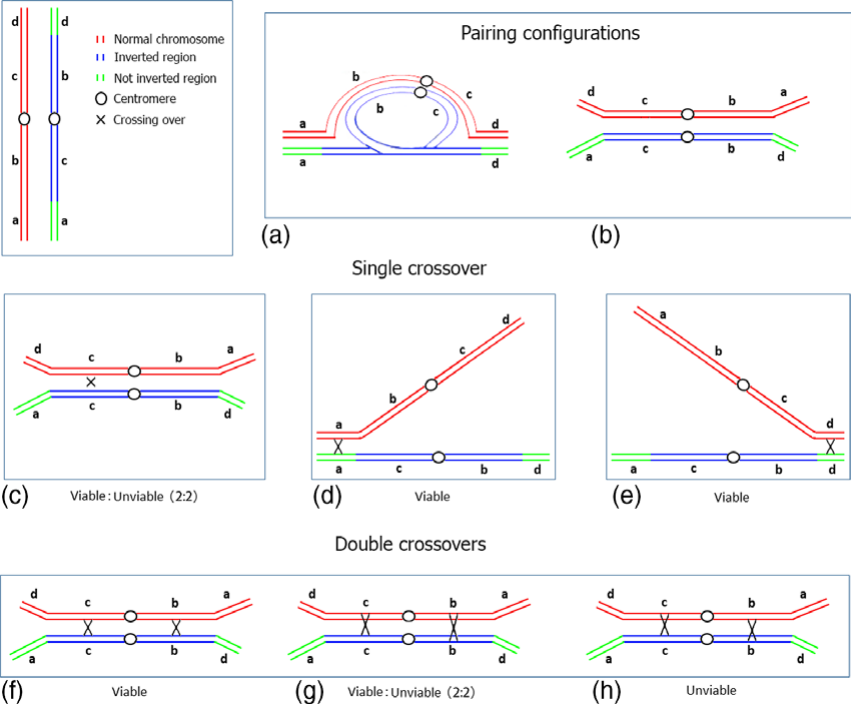
Schematic scenarios of meiotic pairing between chromosomes differing in a pericentric inversion. Pairing of the inverted chromosomal region (homosynapsis) is illustrated as inversion loop (a) or linear with anti‐parallel orientation and unpaired outside regions (b). For simplicity, crossovers (COs) are exemplified in the linear pairing configuration. Single COs within an inversion account for 50% of unviable meiotic products (c). Single (or more) COs in the outside regions do not influence gametic viability (d, e). Two‐strand double COs produce all viable products (f), while three‐strand (g) and four‐strand double COs (h) result in 50% gamete viability and no viable products, respectively.

To examine how the inversion on chromosome 3 affects the recombination rate within the inverted region, we estimated the number of COs in the segment between markers CH3‐3 and CH3‐23 (these markers are close to the breakpoints of the rearrangement). We found that Mctr and Fctr BC1 populations had many cases of single CO events, whereas the mutant populations (Mmut and Fmut) had no single COs (Table 1). This can be explained by the unviability of gametes that carry both deficiencies and duplications of genomic regions. Such a situation will occur when the chromatids are involved in an odd number of COs within the inversion (Figure 4). Gametes, both male and female, that carry these chromatids are thus eliminated by post‐meiotic selection. Conversely, gametes carrying chromatids with an even number of COs are viable (Figure 4).

**Table 1:** Crossover (CO) number on chromosome 3 in the region within inversion (Ch3‐3/Ch3‐23) and the regions outside the inversion (Ch3‐1/Ch3‐2 and Ch3‐24/Ch3‐27) as estimated by single nucleotide polymorphism markers in backcross populations from control (Mctr, Fctr) and mutant (Mmut, Fmut). *P*‐values associated with the H0 hypothesis that control and mutant populations have the same number of COs (chi square)

Region between markers	Mctr			Mmut			*P*‐value	Fctr			Fmut			*P*‐value
	CO = 0	CO = 1	CO = 2	CO = 0	CO = 1	CO = 2		CO = 0	CO = 1	CO = 2	CO = 0	CO = 1	CO = 2	
CH3‐1/CH3‐2	404	10	0	415	3	0	0.07	413	3	0	414	5	0	0.51
Ch3‐3/Ch3‐23	158	222	32	400	0	18	9.45E‐131	238	168	9	415	0	4	2.14E‐66
Ch3‐24/Ch3‐27	375	37	2	370	48	0	0.06	402	14	0	337	82	0	9.93E‐75

**Figure 5:**
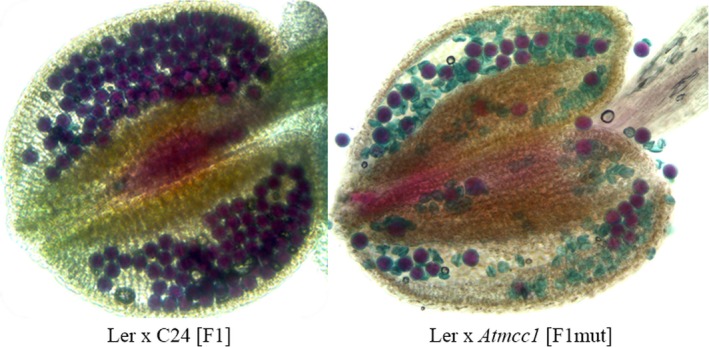
Alexander's staining of pollen grains in anthers of L*er* × C24 [F1] and L*er* × *Atmcc1* [F1mut] plants. Viable pollen is red while unviable pollen is green.

A first observation is that double COs within the inversion were reduced in mutant populations compared with the control (Table 1). A second observation is that COs forming exclusively in the regions outside the inversion have no effect on the viability of post‐meiotic products (Table 1, Figure 4). This post‐meiotic hypothesis is supported by the low pollen viability of F_1_ L*er* × *Atmcc1* that decreased by approximately half comparison with the control F_1_ L*er* × C24 (Figure 5). With these features in mind, we generated a working model of both chromosome pairing and post‐meiotic selection in heterozygotes for the inversion. In considering our model choices, we only found products where the COs were in *just one* of the following three regions: (1) markers CH3‐1/CH3‐2, (2) markers CH3‐3/CH3‐23 or (3) markers CH3‐24/CH3‐27. For instance, individuals simultaneously carrying COs on both terminal regions outside the inversion were not observed. This result suggests that homologs of chromosome 3 pair in only one of the three possible regions as would follow in a linear anti‐parallel pairing scenario. However, more complicated pairing scenarios are possible, for instance involving an inversion loop (Figure 4), as long as the same gamete viability rules are enforced. Our model is characterized by the three unknown probabilities of pairing (one for each region) that add up to one. In addition, we suppose that the gametes have an even number of COs in the inversion, otherwise gamete viability is lost. Lastly, the recombination rates in each interval (arising before selection) are set so that they replicate the rates observed after selection (see details in Experimental procedures). The calibration of the model to the data for male meiosis led to pairing probabilities of, respectively, 0.08, 0.17 and 0.75 in regions 1, 2 and 3 specified above. The same analysis in female meiosis led to the pairing probabilities of 0.06, 0.19 and 0.75, respectively.

Given the specification of our model and its calibration, we were also able to produce simulated gametes and thereby construct associated two‐point linkage heat‐maps for chromosome 3 (Figures 3b and S2b). Those heat‐maps were then compared with the corresponding heat‐maps produced using the experimental data (Figure 3 for male meiosis, Figure S2 for female meiosis). The agreement of the model‐based maps with the experimental‐based maps indicates the relevance of the pairing and post‐meiotic selection hypotheses that we have introduced. Analysis of these data reveals a small difference whereby the model predicts too many COs in the inverted region [lower logarithm of the odds (LOD) scores in the predicted heat maps]. *A posteriori*, this is understandable because we assumed no CO interference in the model in order to be able to calibrate it (see Experimental procedures). That simplification naturally leads to more cases of double COs than occur in the experimental data, as is corroborated in Figures 3 and S2. Note that this effect is stronger in female meiosis, as expected, since CO interference is significantly stronger in female than in male meiosis in Arabidopsis (Giraut *et al*., 2011). The higher female CO interference could also explain the particularly low number of double COs occurring in the region within the inversion (Table 1). The higher number of COs located in the region outside markers CH3‐24 and CH3‐27 could be the compensation effect that guarantees the obligatory CO on chromosome 3.

### Chiasmata decrease in chromosome 3 in male meiosis

**Figure 6:**
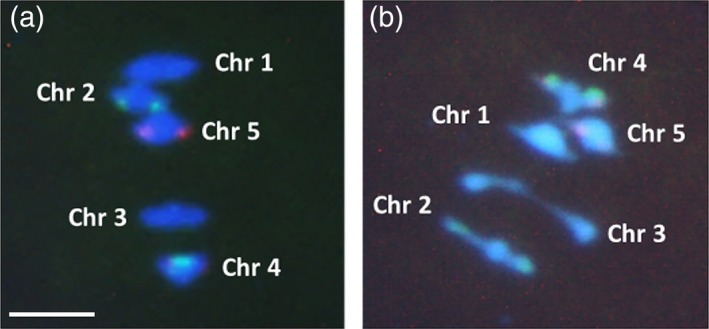
Fluorescence *in situ* hybridization (FISH) from F_1_ L*er* × C24 (a)and L*er* × *Atmcc1* (b) at metaphase I in male meiosis. The FISH is performed with 5S rDNA (red) and 45S rDNA (green) probes. Blue is 4′‐6‐diamidino‐2 phenylindole stained DNA. Scale bar: 5 μm.

Because of post‐meiotic selection, the genetic data do not provide a *direct* estimation of the CO number during meiosis in chromosome 3 in *Atmcc1*. For this reason, we evaluated the number of chiasmata during metaphase I of male meiosis in F_1_ L*er* × *Atmcc1* in comparison with F_1_ L*er* × C24 (as the control) according to the cytological method described by Sanchez‐Moran *et al*. (2001). For chromosome 3, distinguished from chromosome 1 by size, there was a significant decrease in the number of chiasmata per cell in L*er* × *Atmcc1* versus the control (1.11 versus 1.69, Fisher's test <0.001) (Figure 6). Analysis of bivalent configurations revealed an increase in rod bivalents (where the chiasma is restricted to a single arm) in L*er* × *Atmcc1* (88%; *n* = 60 cells) when compared with L*er* × C24 (30%; *n* = 36 cells) (χ^2^ = 2.268 with Yates correction; *P* = 0.01). This change can be interpreted within our model: if pairing in the heterozygote mutant occurs within one segment it is rare for a bivalent to have two COs. This interpretation also justifies the deficit in double‐CO events observed at the gametic level (Table 1).

### The number of COs on the rest of the genome reveals the IC

**Figure 7:**
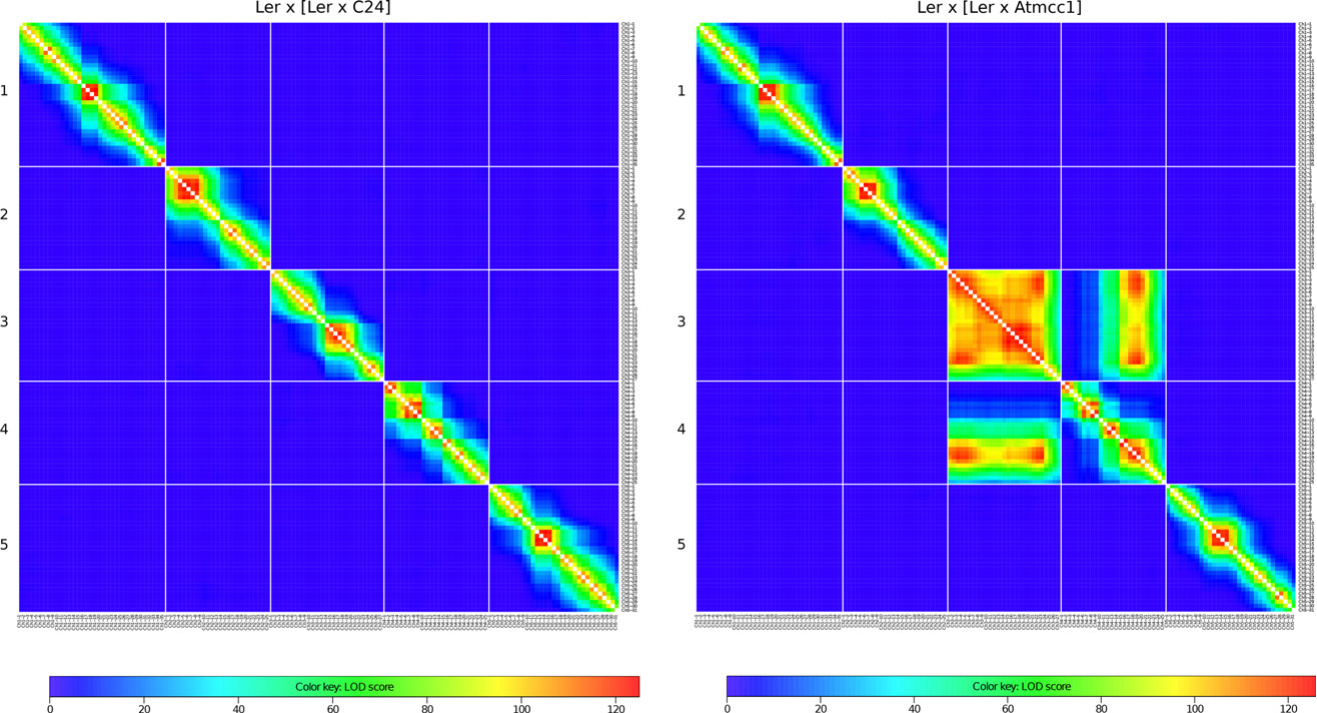
Genome‐wide heat maps of logarithm of the odds (LOD) scores for pairwise linkage in male meiosis. Left: control population showing the standard structure of LOD decreasing as one goes away from the diagonal and no significant LODs between markers on different chromosomes. Right: mutant population showing the standard structure for chromosomes 1, 2 and 5 but abnormal behavior between chromosomes 3 and 4.

After describing the impact of the inversion on COs in chromosome 3 (the intrachromosomal effect), we tested whether the reduction in recombination in chromosome 3 is compensated by increased recombination in the other chromosomes (the IC). Although the construction of individual genetic maps gave the same order as the physical one for chromosomes 1, 2, 4 and 5, abnormal linkage was found between chromosomes 3 and 4 in both the male and female crosses involving the mutant, as shown in the heat maps (Figure 7 for male meiosis and Figure S6 for female meiosis). Such an anomalous linkage suggests a structural rearrangement involving chromosomes 3 and 4. Looking at the Marey map of chromosome 4, recombination appeared suppressed in a region of the south arm (Figure 2 for male meiosis and Figure S1 for female meiosis). Furthermore, we observed (particularly in Mmut) a conspicuous spike in CO frequency occurring in the north arm of chromosome 4, which is probably a consequence of the intrachromosomal compensation effect (Figure 2 for male meiosis and Figure S1 for female meiosis). Thus, the average number of COs in chromosome 4 was identical (1.51) in Mmut and Mctr and similar in Fmut and Fctr (1.28 versus 1.25). Since the recombination products relative to chromosome 3 are involved in post‐meiotic selection, it is possible that something analogous occurs for chromosome 4 due to the IC linkage between chromosomes 3 and 4. For this reason, we omitted chromosomes 3 and 4 in the following analyses.

We thus examined CO statistics in chromosomes 1, 2 and 5. Since these chromosomes showed no abnormal linkage (Figure 7) we can reasonably assume that they are not affected by post‐meiotic selection and therefore we can use their genetic data as an estimation of CO number. When comparing the mutant and control, pairwise comparisons of genetic lengths reveal a significant increase of COs in the mutant for chromosomes 1, 2 and 5 in male meiosis (*P* < 10^−7^ when pooling all three chromosomes) (Table 2). Indeed, in the mutant, chromosomes 1, 2 and 5 have more COs by 13%, 21% and 22%, respectively. Such an increased number of COs in these chromosomes associated with CO loss in chromosome 3 is a signature of the IC effect that keeps the total number of COs per cell unchanged. We can argue that chromosomes 1, 2 and 5 are compensating for the recombination suppression occurring in chromosome 3 by forming additional COs. To determine whether the IC effect is targeted to particular regions, we performed statistical tests for pairwise comparisons of the recombination landscapes of chromosomes 1, 2 and 5 between control and mutant. This analysis did not reveal any significant difference (Figure S7). In female meiosis, the IC effect is much less clear: the trend in female meiosis is for the genetic lengths in Fmut to be higher than in Fctr, but the effect is not statistically significant (Table 2). We hypothesized that the IC effect in the female is negligible due to the strength of CO interference that resists other factors facilitating the formation of further COs.

**Table 2:** CO numbers in chromosomes 1, 2 and 5 as estimated by SNP markers in backcross populations from control (Mctr, Fctr) and mutant (Mmut, Fmut)

	Map size (cM)		Recombination rate (cM/Mbp)		*P*	Map size (cM)		Recombination rate (cM/Mbp)		*P*
	Mctr	Mmut	Mctr	Mmut		Fctr	Fmut	Fctr	Fmut	
Chr 1	123.13	141.37	4.05	4.65	0.02	79.64	83.84	2.62	2.76	0.50
Chr 2	74.61	94.93	3.79	4.82	0.001	62.80	60.32	3.19	3.06	0.65
Chr 5	110.99	141.47	4.12	5.25	8.08E‐05	68.86	75.12	2.55	2.79	0.29
ALL	102.91	125.92	3.98	4.90	8.19E‐07	70.43	73.09	2.79	2.87	0.62

To rule out the possibility that CO increases on chromosomes 1, 2 and 5 could be due to the mutated *Atmcc1* allele, we used real‐time RT‐qPCR to quantify the transcript of *AtMCC1* in the L*er* × *Atmcc1* F_1_ mutant compared with L*er* × C24 F1 as the control (Figure S8). As expected, *AtMCC1* was overexpressed in the F_1_ mutant (1.6 fold). A similar value for expression difference was reported in homozygous *Atmcc1* versus C24 (control) by Perrella *et al*. (2010). This allows us to exclude the *Atmcc1* allelic IC effect.

To understand whether the additional COs in male meiosis could be associated with a change in the interference, we measured the interference strength by fitting the segregation data to the so‐called two‐pathway gamma model using CODA software (Gauthier *et al*., 2011). Following what was done for the recombination rates, we restricted the analysis to chromosomes 1, 2 and 5. There is a trend whereby the mutant generally has lower interference strength than the control (Table S2). However, the low number of pairwise comparisons (six, coming from the two sexes for each of the three chromosomes) prevented us from rejecting the null hypothesis of no trend (one‐sided *P*‐value of 0.17 when using the pooled data).

## Discussion

In this study, we have shown in Arabidopsis that the reduced ability of a single chromosome to recombine in meiosis, following changes in its structure, produces significant effects on the other chromosomes. In our case, the structural rearrangement is a large pericentric inversion involving chromosome 3. The inversion is associated with the *Atmcc1* mutation previously characterized in the homozygous line (Perrella *et al*., 2010). The genetic maps obtained in this work using 143 SNP markers in heterozygous *Atmcc1* populations of over 400 individuals provide evidence of CO loss in chromosome 3 in both male and female meiosis. As expected, post‐meiotic selection operates against unbalanced gametes carrying deficiencies/duplications of genomic regions that are usually produced from bivalents carrying both single COs and three‐ or four‐strand double COs localized within the inverted region (Burnham, 1962). In our mutant populations post‐meiotic selection is relatively high, as indicated by the fact that single COs in the inverted region of chromosome 3 were never observed, a feature treated in our model for post‐meiotic selection. For male gametes, this selection is confirmed experimentally by pollen viability analysis. The significant post‐meiotic selection can be explained by the fact that the large size of the inversion is related to a high incidence of COs in that region and consequently to a large proportion of unbalanced/unviable meiotic products. This is in line with the existence of a relationship between the size of the inversion and the frequency of unbalanced gametes as reported for other organisms (Burnham, 1962). Because post‐meiotic selection is strong, genetic data cannot provide an estimation of CO number for chromosome 3 in *Atmcc1*. However, the chiasma counts in heterozygous *Atmcc1* allowed us to establish that CO suppression occurs in chromosome 3 during male meiosis. In particular, we measured 1.11 chiasmata per cell in chromosome 3, corresponding to a decrease of 34% compared with the control. Obligate CO was maintained, thereby ensuring the correct segregation. In previous literature, the number of chiasmata for chromosome 3 is reported to range from 1.72 to 2.04 in different Arabidopsis ecotypes (Sanchez‐Moran *et al*., 2002), including C24 and L*er* which were used in this work. Accordingly, 2.14 COs, on average, were found on chromosome 3 by Giraut *et al*. (2011) in a Col × L*er* cross using genetic data. Based on comparable chiasma numbers in chromosome 3 between homozygous *Atmcc1* (1.68) and the wild type (1.54) previously published by our group (Perrella *et al*., 2010), the present study suggests that structural rearrangement is the only cause of the decreased recombination observed in heterozygous *Atmcc1*. The reduction/suppression of COs within inversions is well documented across several organisms (Dresser *et al*., 1994; Fransz *et al*., 2016). Different mechanisms are invoked to explain this CO suppression such as non‐homologous synapsis (hetero‐synapsis) (Torgasheva and Borodin, 2010) and asynapsis (Kirkpatrick *et al*., 2012) between the normal and inverted regions. However, in our work recombination events such as double COs are detected in chromosome 3, suggesting that homosynapsis occurs in the rearranged region. Moreover, homosynapsis is indirectly confirmed by post‐meiotic selection. As reported in other species (Anton *et al*., 2005), homosynapsis can imply the formation of an inversion loop or, alternatively, the pairing of the region with inversion in the anti‐parallel orientation associated with pairing failure of the outside regions. The latter could be prominent with respect to the inversion loop based on the observations of CO distribution in chromosome 3 of individual plants. Indeed, COs located in the inverted region never coexist with COs in the outside regions. Furthermore, COs on both outside regions do not occur in our plant material. This pairing behavior can account for the deficit of COs on chromosome 3. Looking at the total number of COs, we confirmed that CO loss within an inversion was not fully counterbalanced by an increase of COs in outside regions on chromosome 3. In diverse Arabidopsis structural rearrangements (inversions and deletions) induced by gamma rays. Ederveen *et al*. (2015) found that CO decreases in the rearranged regions was generally compensated for by increases in COs on the unaltered part of the same chromosome. Similar behavior was found in individuals of *C. elegans* carrying an inversion (Zetka and Rose, 1992). We speculate that the differences between our case and those described in the literature are related to the size of the structural rearrangement**.** Indeed, the inversion covers 80% of the chromosome length in our material compared with a maximum value of 39% found by Ederveen *et al*. (2015). This implies that in *Atmcc1* the regions outside the inversion are restricted in size. The size of these regions, lying between the inversion boundaries and the telomeres, may constrain the opportunities for CO events. Our observation that COs occur preferentially in the largest outside region (the south arm) is consistent with this hypothesis. Besides the size, the chromatin structure of these regions can influence CO formation. For example, in *Drosophila*, recombination suppression was estimated to extend 2.5–3 Mbp beyond the heterozygous inversion boundaries (Stevison *et al*., 2011) and similar observations were reported in other organisms (White and Morley, 1955; Strasburg *et al*., 2009). Regarding telomeres, in budding yeast the 20 kbp region adjacent to the telomeres exhibits a significantly lower recombination rate (Pan *et al*., 2011). In Arabidopsis, the telomeres are estimated to be 2–5 kbp long (Vaquero‐Sedas and Vega‐Palas, 2014) but recombination data are not available in these domains due to resolution limits of the tools used for analyzing recombination events.

The most striking finding of the present study was that the partial failure of chromosome 3 to recombine was coupled with an increase in CO frequencies on the other chromosomes in male meiosis. The mutated *Atmcc1* allele does not seem to be responsible for the increase in COs on chromosomes 1, 2 and 5. Indeed, the homozygous *Atmcc1* displayed unchanged COs on chromosome 5 and reduced COs on chromosomes 1 and 2 (Perrella *et al*., 2010). In addition, we did not observe any difference in the expression level of *AtMCC1* in the two allelic forms of *Atmcc1*, indicating that its expression is not responsible for the recombination behavior observed in our inversion heterozygous populations. Therefore, we argue that COs are increased on chromosomes 1, 2 and 5 to counterbalance the loss of COs due to the rearrangement on chromosome 3. This in turn maintains the total number of COs per cell relatively unchanged. The IC effect reported here is similar to the phenomenon described in female meiosis in *Drosophila* (Schultz and Redfield, 1951) and maize (Bellini and Bianchi, 1963; McKinley and Goldman, 1979). Since an increase in COs can be expected to be associated with changes in interference, we also performed an interference analysis. We detected lower interference in the mutant, but this was not statistically significant. This is similar to what was found in Drosophila where the presence of heterozygous inversion induced the IC effect but not weakening of interference (Lucchesi and Suzuki, 1968). To understand the molecular mechanisms underlying the IC effect, *Drosophila* mutants carrying inversions have been investigated (Joyce and McKim, 2011; Crown *et al*., 2018). In these works, it was suggested that heterozygous inversions cause a discontinuity in the alignment between homologs that can induce defects in axis structure. Consequently, the chromosome pair is destabilized over time, thereby inducing a pachytene delay that increases the chance of DSBs becoming COs at the expense of NCOs. In this model, the response by all chromosomes to maintain the expected number of COs per cell is mediated by the AAA+ATPase PCH2 (Joyce and Mckim, 2010; Joyce and McKim, 2011) without increasing the number of DSBs (Crown *et al*., 2018). In *C. elegans*, asynapsis of the X chromosome resulted in an IC response on autosomes (Carlton *et al*., 2006). In this case, IC is associated with a delay in meiotic progression, similar to what happens in flies, but requiring additional DSBs and their resolution as COs. In both flies and worms, IC is explained as an effect of the disruption of the normal timing of meiotic prophase events. To ensure the expected number of COs per cell, we can speculate from our results that Arabidopsis also has a window of opportunity for CO formation through either initiation of new DSBs or, more likely, NCOs being redirected into COs. In this study, we found that the IC effect is not evident in female meiosis. The extent of the IC effect, if any, would be quite modest simply because the high CO interference constrains any increase in CO number beyond the minimum value imposed by CO assurance. Indeed, COs in Arabidopsis female meiosis are typically limited to barely more than one CO per chromosome pair, about two‐thirds of the number of COs observed in males (this work; Giraut *et al*., 2011). Lloyd and Jenczewski (2019) propose that the major differences between the sexes for CO number, distribution and interference strength are due to a difference in synaptonemal complex length between the male and female meiosis.

In conclusion, we discovered that the IC effect is a phenomenon arising in male recombination in Arabidopsis. In natural populations, inversions are recognized as the most effective force in reducing COs. However, the effect of heterozygous inversions not only results in a local suppression of COs but also leads to a genome‐wide increase in genetic exchanges.

## Experimental Procedures

### Plant material

The *Arabidopsis thaliana* genotypes that were used in all of the experiments described in this work include the *Atmcc1* mutant (Perrella *et al*., 2010) and the ecotypes C24 (Ishitani *et al*., 1997) and L*er*. The *Atmcc1* mutant was previously isolated from an activation tagging population (Perrella *et al*., 2006) obtained after floral transformation by *Agrobacterium tumefaciens* with the binary vector pSKI015 (Koiwa *et al*., 2002). The homozygous line of *Atmcc1* was characterized for male meiosis (Perrella *et al*., 2010) and expression profiling (Barra *et al*., 2012). C24 is the genetic background of *Atmcc1* and is used in this study as a wild type. C24 refers to a homozygous line for a T‐DNA region very close to the marker AtGAPab in chromosome 3 (Ishitani *et al*., 1997; Gong *et al*., 2002). *Atmcc1* has an additional T‐DNA insertion very close to the At3g02980 locus on the long arm of chromosome 3, near the north telomere (Figure S9). The accession of L*er* (213AV) was kindly given by the Centre de Resources Biologiques at the Institut Jean Pierre Bourgin, Versailles, France (http://publiclines.versailles.inra.fr/). Plants were grown in controlled growth chambers with 16 h/8 h of light/dark at 22°C/18°C.

### Construction of the recombinant population

L*er* was crossed with C24 and *Atmcc1* to obtain two sets of F_1_ progeny (Figure 1). About 50 F_1_ hybrids were backcrossed with L*er* using F_1_ plants as either the male or the female parent to obtain four BC1 populations. For each BC1 population, about 700 seeds were sown *in vitro*. Subsequently, after 2 weeks, 430 seedlings transferred in pots were grown in the controlled growth chambers for 3 weeks.

### Genomic DNA extraction

Extraction of DNA was performed as described by Giraut *et al*. (2011) with some modifications. Two hundred milligrams of green tissue from each BC1 plant was collected, freeze‐dried, lyophilized and ground in 96‐well plates, closing the wells hermetically with plastic caps. One milliliter of Extraction Buffer [2‐amino‐2‐(hydroxymethyl)‐1,3‐propanediol (TRIS) pH 8 0.1 m, EDTA 50 mm, NaCl 0.5 m, SDS 1.25%, polyvinylpyrrolidone 40000 1%, sodium bisulfite 1%, pre‐warmed at 65°C] was then added to each well and the plates were incubated at 65°C for 30 min. 300 microliters of a solution of cold 60% potassium acetate (KAc) 3M and 11.5% glacial acetic acid was added to each well. The plate was sealed with a Thermowell film (Corning, https://www.corning.com/emea/en.html), shaken gently and placed on ice for 5 min. After centrifugation in a A‐4‐62 rotor (Eppendorf, https://www.eppendorf.com/) at 3220 ***g*** for 10 min at 4°C, 800 ml of the supernatant was transferred to a clean DeepWell plate and 1 ml of CGE buffer (1/3 guanidine hydrochloride 7.8 m, 2/3 ethanol 96%) was added per well. Six hundred milliliters of the mixture was filtered with a Whatman Unifilter 800 GF/B plate placed on a DeepWell plate (Greiner Bio‐One, https://www.gbo.com/) and centrifuged for 2 min at 5806 ***g*** in a Nr 09100F rotor (Sigma, https://www.sigmaaldrich.com/) at room temperature (25°C). The flow‐through was discarded. This step was repeated twice. The membrane was washed twice by adding 500 ml of washing buffer (37% aqueous solution, 63% ethanol 96%; aqueous solution = KAc 160 mm, TRIS HCl pH 8 22.5 mm, EDTA 0.1 mm) and then centrifuged for 2 min at 5806 ***g*** at room temperature. The DNA was eluted with 70 ml of H_2_O by centrifugation for 2 min at 363 ***g*** at room temperature. This step was repeated twice. Then RNAse A was added to 0.5 mg ml^−1^ and the DNA concentration was determined using a Quant‐iT dsDNA BR assay kit (Invitrogen, https://www.thermofisher.com) with an ABI 7900HT real‐time PCR system (Applied Biosystems, www.thermofisher.com).

### Genotyping SNP markers

A genome‐wide set of 146 SNP markers polymorphic between C24 and L*er* was selected using the POLYMORPH website (http://polymorph.weigelworld.org/cgi-bin/webapp.cgi?page=Home;project=MPICao2010) (Clark *et al*., 2007; Zeller *et al*., 2008). Twenty per cent of SNPs were validated by Sanger sequencing in our lab. The physical position of each marker was verified using the SEQVIEWER tool from http://www.arabidopsis.org. Evenly distributed informative SNPs with an average spacing of 0.8 Mb were used for genotyping BC1 populations. For each marker, a total of 1680 plants were genotyped by LGC Genomics through KASP™ technology (https://www.lgcgroup.com/). The DNA was purified with Whatman Unifilter plates and quantified to a master dilution of 5 μg total DNA. The DNA was transferred into appropriate microtiter plates using a LGC RepliKator™ robot (https://www.biosearchtech.com/). The final DNA concentration was 50 ng μl^−1^ per well of sample and the KASP procedure was carried out by LGC Genomics following a standard KASP assay procedure (He *et al*., 2014). Alleles were called by KBioscience (LGC Genomics Group) (Figure S10). Each SNP was checked using SNPviewer2 software and rescored whenever any error was observed in the clustering of the homozygous and heterozygous genotypes. Three SNPs were removed from the final dataset due to bad KASP calling. Twenty‐one plants with more than 50% of uncalled alleles were discarded. High‐resolution melt analysis was also performed in some ambiguous cases according to Terracciano *et al*. (2013). The 143 remaining SNP markers are listed in Table S1 and raw genotyping data are reported in Table S3.

### Analysis of segregation distortion

To test whether any deviation from normal segregation (1:1) occurs in BC1 populations, the number of plants with the C24 allele at each marker locus was counted considering that the frequency of each allele should not deviate from 50% plus a delta value calculated as standard deviation according to Giraut *et al*. (2011). No significant segregation distortions were found in the populations except for a few slight divergences that do not bias our estimate of genetic lengths (Figure S11).

### Construction of genetic maps, Marey maps and linkage heat maps

The order of the markers was taken from the Weigel database (Clark *et al*., 2007; Zeller *et al*., 2008) for L*er*. Nevertheless, for completeness, we also determined it *ab initio* based on genetic linkage only. In the case of chromosome 3 in crosses involving the mutant, we found that the *ab initio* orders were ambiguous, reflecting the presence of the inversion. In contrast, for all other chromosomes, the *ab initio* orders were unambiguous and identical to those in the reference genome. For each BC1 population, genetic lengths between adjacent markers were calculated using Kosambi's function. Based on these genetic maps, we computed the associated Marey maps. Recombination rates were obtained as the first derivative of the curve representing genetic versus physical positions. Finally, linkage heat maps were produced by calculating the LOD for linkage for each pair of markers, whether they were on the same chromosome or not. Two‐point linkage analyses were performed using CarthaGene software (de Givry *et al*., 2005) called from R scripts. Such heat maps can provide signatures of structural rearrangements within chromosomes and even reveal unexpected linkage between different chromosomes.

### Modeling of homolog pairing and post‐meiotic selection in heterozygotes for the inversion

Since we found only cases where the COs were in *just one* of the three regions, namely (1) markers CH3‐1/CH3‐2, (2) markers CH3‐3/CH3‐23 and (3) markers CH3‐24/CH3‐27, we hypothesized that in each meiosis one and only one of those three regions could pair. Our model assigns to each region a probability of pairing (P1, P2, P3). These quantities are *a priori* unknown and have to be calibrated based on the data as they correspond to unobservable quantities that arise before any selection. Once one of the regions is paired, COs can form within that region, and we model this using a recombination rate for each interval between adjacent markers. This process generates gametic products that are then subject to post‐meiotic selection: only gametes with an even number of COs within the inversion are kept as viable. The main consequence for our modeling of this selection is that the observable recombination rates are not the ones specified before selection. Interestingly, it is possible to relate these rates by an explicit formula if and only if one assumes that there is no CO interference. That allowed us to perform a scan of the P1, P2 and P3 values (corresponding to the exploration of a two‐dimensional grid for two unknowns, since P1 + P2 + P3 = 1), and for each trial point the recombination rates were set to their correct values in order to reproduce the rates observed in the mutant. For each value of P1, P2 and P3, we computed the expected frequencies of pairing for each region *after selection*. The function ‘optim’ of R was used to determine the two parameter values giving the best agreement between observed and simulated frequencies of pairing in each region and the probability of having zero COs (in which case the pairing region is unknown).

### Crossovers and distribution of number of COs per chromosome

For each BC plant, the number of COs was calculated by scoring allele changes across single chromosomes. The average number of COs per chromosome pair was estimated by dividing the total number of COs for that chromosome by the number of plants in the population and multiplying by 2.

### Real‐time RT‐qPCR analysis

Total RNA was extracted from young leaves of L*er* × *Atmcc1* and L*er* × C24 F_1_ plants using the RNeasy Plant Mini Kit (Qiagen, https://www.qiagen.com/) and treated with DNase I (Invitrogen). To obtain complementary DNA, SuperScript III Two‐Step RT‐PCR and Oligo(dT)12–18 (Invitrogen) were used following the manufacturer's instructions. The gene‐specific primers designed using Primer Express software, v.2.0 (Applied Biosystems), were AAACCGTGGAATCGCAATGT (forward) and TCGCAGCATTGTTGTGTCC (reverse). Real‐time RT‐qPCR was carried out using Applied Biosystems 7900 HT instrument (Applied Biosystems) and Power SYBR Green PCR Master Mix (Applied Biosystems). The *ADENOSINE PHOSPHORIBOSYL TRANSFERASE1* (*APT1*) gene was used as the reference (primer sequences are ATTTGTTCCCATGAGGAAGCC and CACCTACGTGCATCTCAATCGT as forward and reverse, respectively).

### Cytological analysis of chiasmata and pollen viability

Chiasmata were counted at metaphase I in male meiosis which was investigated using the spreading technique described by Ross *et al*. (1996) with some modifications. Briefly, floral buds, previously fixed in ethanol;acetic acid (3:1 v/v), were rinsed in freshly made ethanol;acetic acid (3:1 v/v), followed by citrate buffer. Then buds were digested in an enzyme mixture consisting of 0.3% (w/v) cellulase and 0.3% (w/v) pectolyase for 1 h 15 min at room temperature, followed by 30 min at 37°C. After digestion, the buds were rinsed in citrate buffer. One or two whole buds were then transferred on a slide and were macerated with a needle in 10 μl of 60% acetic acid on a hot‐plate (45°C) for 30 sec to release meiocytes. The meiocytes were then fixed with ice‐cold ethanol:acetic acid (3:1 v/v). The slides were air dried and stained using 4′‐6‐diamidino‐2 phenylindole. Slides were analyzed by fluorescence microscopy. Chiasmata were recorded at metaphase I in pollen mother cells after fluorescence *in situ* hybridization following the method of Sanchez‐Moran *et al*. (2001). Pollen viability was assessed on anthers from five open flowers using Alexander's staining (Alexander, 1969).

### Next‐generation sequencing and bioinformatics

We performed NGS on the *Atmcc1* line, used as the parent of F_1_ in this study. The NGS, including sample quality control, was carried out by Genomix4life (https://www.genomix4life.com/). Indexed libraries were prepared from 1 μg/ea purified DNA with a TruSeq DNA Sample Prep Kit (Illumina, https://www.illumina.com/) according to the manufacturer's instructions. Libraries were quantified using an Agilent 2100 Bioanalyzer (Agilent Technologies, https://www.agilent.com/) and pooled such that each index‐tagged sample was present in equimolar amounts, with a final concentration of the pooled samples of 2 nm. The pooled samples were subject to cluster generation and sequencing using an Illumina NextSeq 500 System (Illumina) in a 2 × 150 paired‐end format at a final concentration of 1.8 pmol. The raw sequence files generated (.fastq files) underwent quality control analysis using FastQC (http://www.bioinformatics.babraham.ac.uk/projects/fastqc/). About 26.5 million paired reads of 150 bp were produced, of which 98.1% mapped on the *A. thaliana* reference genome, corresponding to an estimated coverage of 32×. As a control, C24 whole‐genome resequencing data were downloaded from https://1001genomes.org/data-center.html. Both datasets were processed as follows. Trimming and clipping were performed with BBDuk (https://jgi.doe.gov/data-and-tools/bbtools/) setting a minimum base quality of 25 and a minimum length of 35. Trimmed reads were mapped against the TAIR10 *A. thaliana* reference genome with BWA (http://bio-bwa.sourceforge.net/). Duplicated reads were removed with Picard tools (https://broadinstitute.github.io/picard/), whereas those with a mapping quality lower than 30 were removed with samtools (http://samtools.sourceforge.net/). A combination of bash commands and samtools allowed us to extract reads that were not properly paired (‐F 3 samtools option) with an insert size higher than 2000 bp, i.e. corresponding to the reads supporting a putative inversion, mapping in the positions between the genetic markers Chr3‐2 (Chr3:597345) and Chr3‐3 (Chr3:1484401) and Chr3‐23 (Chr3:20283184) and Chr3‐24 (Chr3:21164570). Finally, bedtools intersect (https://bedtools.readthedocs.io/en/latest/) was used to extract the regions present only in *Atmcc1* and supporting the inversion.

## Author contributions

PT conducted genotyping experiment and analyzed data, MF and OCM performed map construction and modeling, RAC analyzed NGS data, GC performed cytological analysis, RP constructed recombinant populations, RP and AE supported the genotyping experiment, FMC and CC conceived and supervised this study, PT, MF, OCM, FMC and CC wrote the manuscript.

## Data statement

All data referred to are included in the manuscript or supplementary materials of this manuscript.

## Conflict of interest

The authors declare no conflict of interest.

## Supporting information


**Figure S1.** Relationship between physical and genetic positions of the markers on each chromosome, and corresponding recombination rates in the female control and female mutant linkage maps.

**Figure S2.** Heat maps for the two‐point linkage logarithm of the odds scores on chromosome 3 with the inversion in female meiosis.

**Figure S3.** Genome browser visualization of the reads supporting the pericentromeric inversion in *Atmcc1*.

**Figure S4.** Genome browser visualization of the read coverage in the control C24.

**Figure S5.** Schematic view of chromosome 3 in control and mutant.

**Figure S6.** Genome‐wide heat maps of logarithm of the odds scores for pairwise marker linkage in female meiosis.

**Figure S7.** Illustration of the statistical test used to compare recombination landscapes between control and mutant populations for chromosomes 1, 2 and 5.

**Figure S8.** Real‐time RT‐qPCR of *AtMCC1* transcript in leaf of L*er* × *Atmcc1* F_1_ mutant plants compared with L*er* × C24 F_1_ control plants.

**Figure S9.** Schematic representation of chromosome 3 indicating some random loci on short and long arm, centromere and T‐DNA insertions in C24 and in *Atmcc1*.

**Figure S10.** Example of KASP genotyping output.

**Figure S11.** Frequency of C24 allele (in orange) at each marker per chromosome in the mapping of the Mctr population.

**Table S1.** List of single nucleotide polymorphisms used for genotyping.

**Table S2.** Interference strength measured by fitting the data to a two‐pathway Gamma model.

**Table S3.** Genotyping scores of the recombinant BC1 populations.
