# Supplementary figures and images for: Recombination suppression in heterozygotes for a pericentric inversion induces the interchromosomal effect on crossovers in Arabidopsis
Source: Plant J. 2019 Oct 7;100(6):1163–75. doi: 10.1111/tpj.14505 (PMC6973161; doi:10.1111/tpj.14505)

Female chr 1

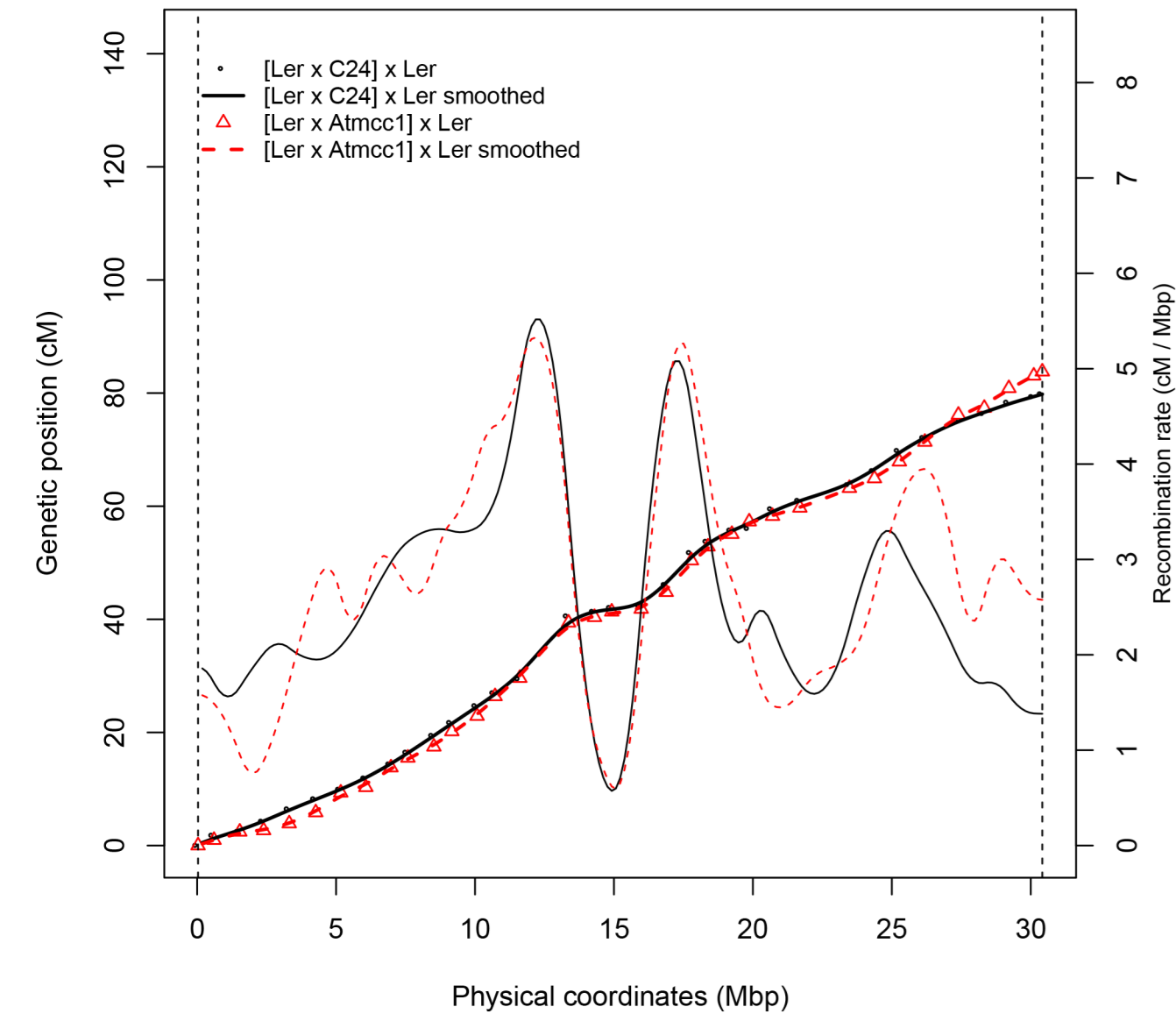

Female chr 2

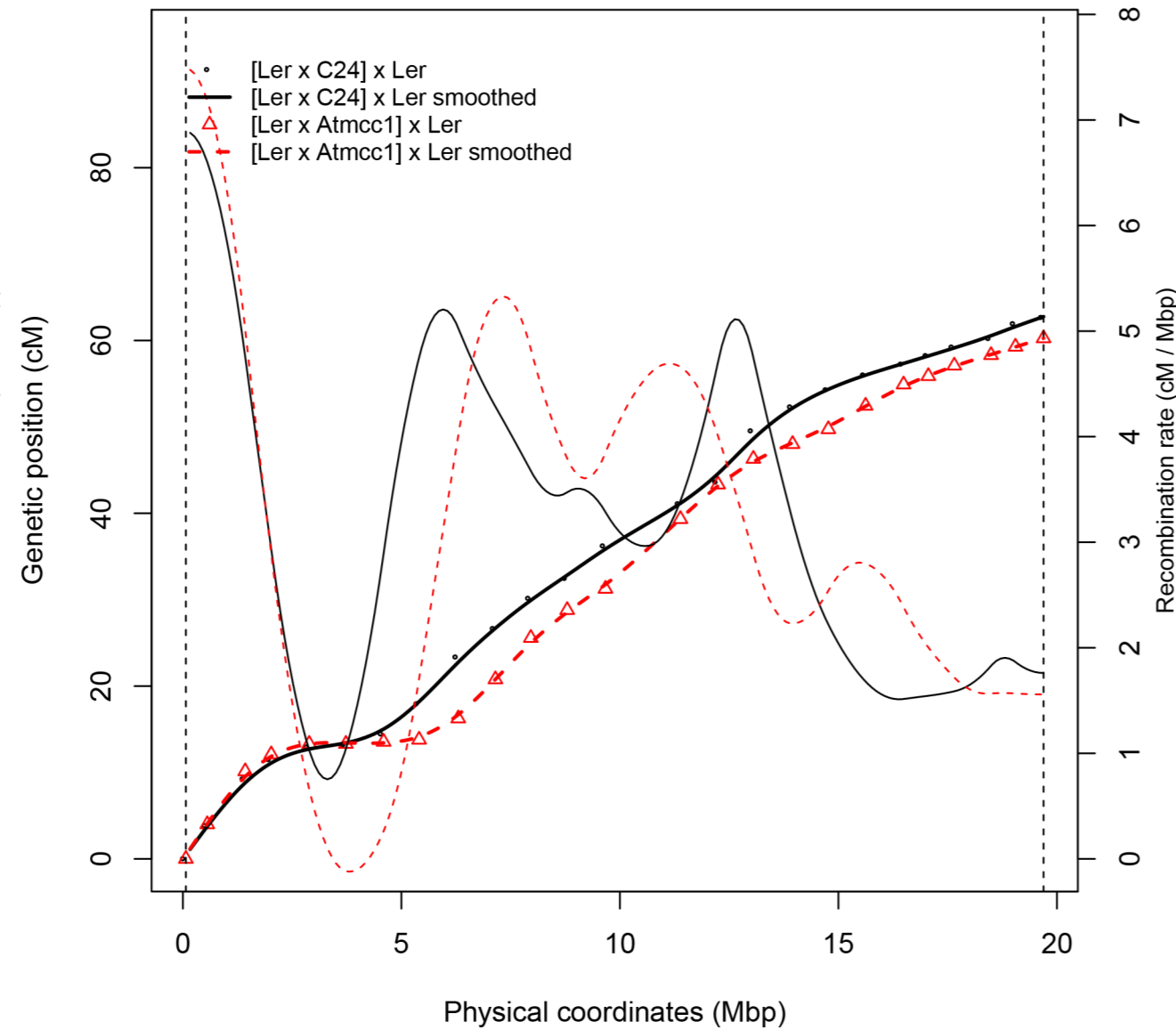

Female chr 3

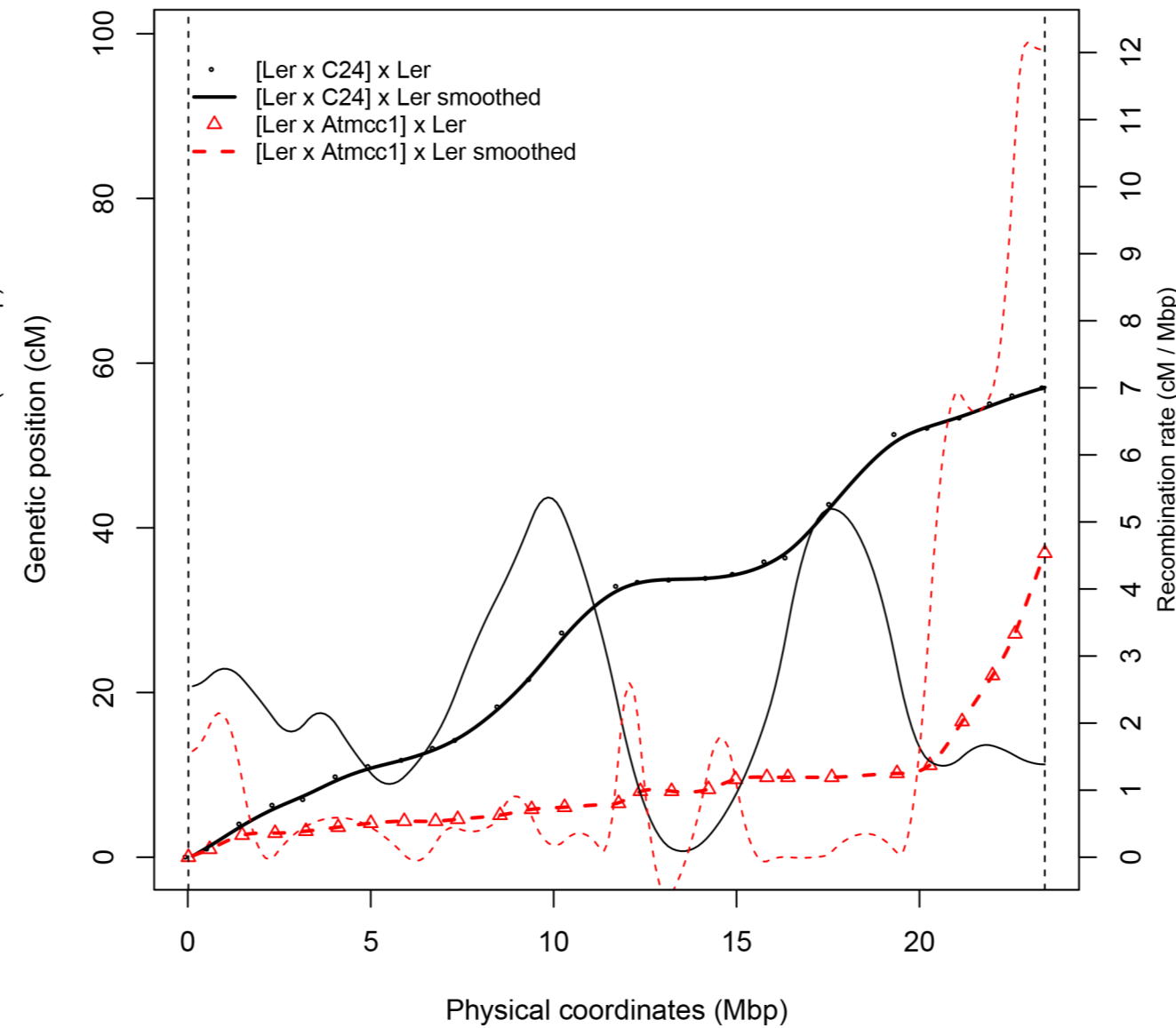

Female chr 4

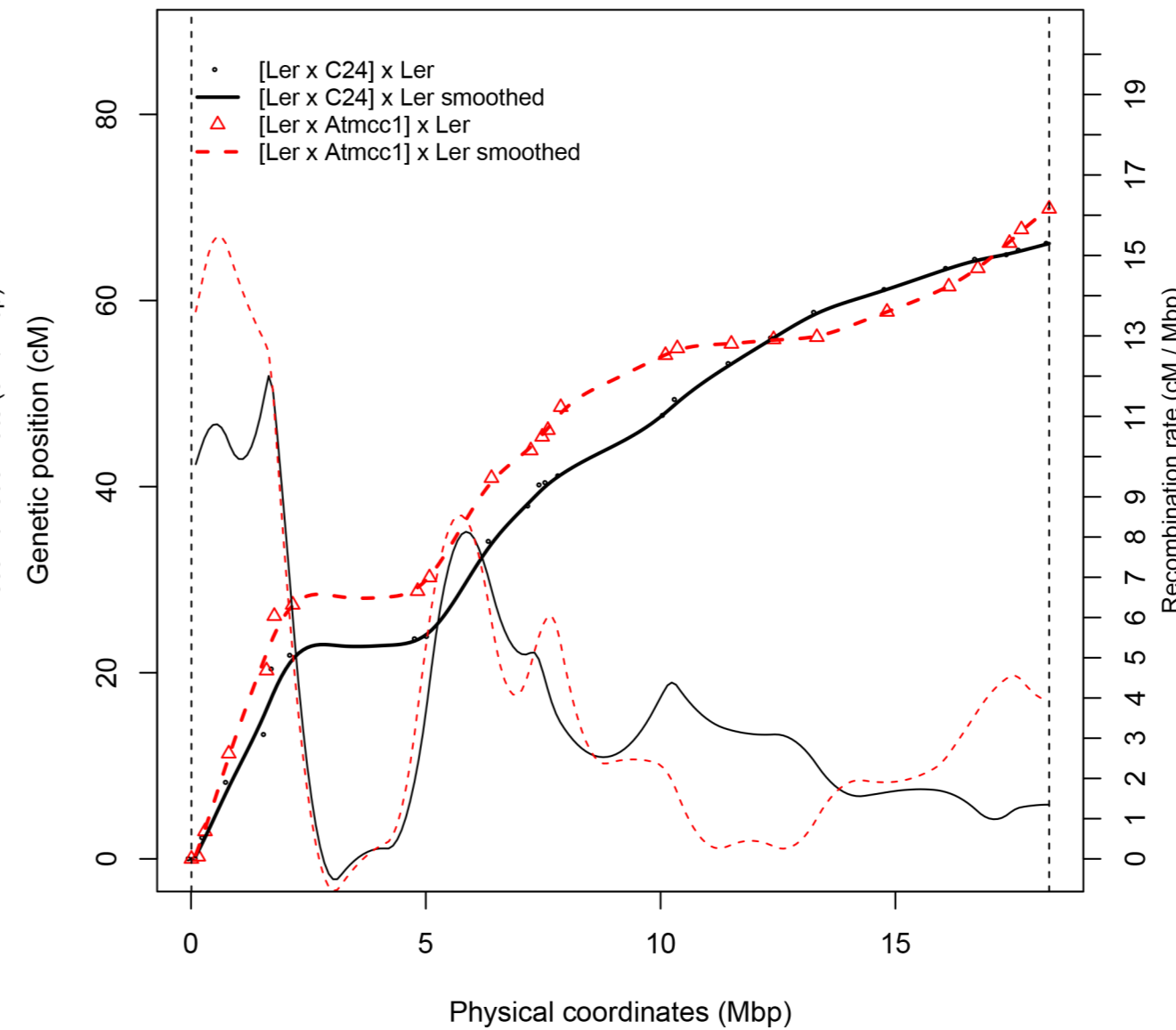

Female chr 5

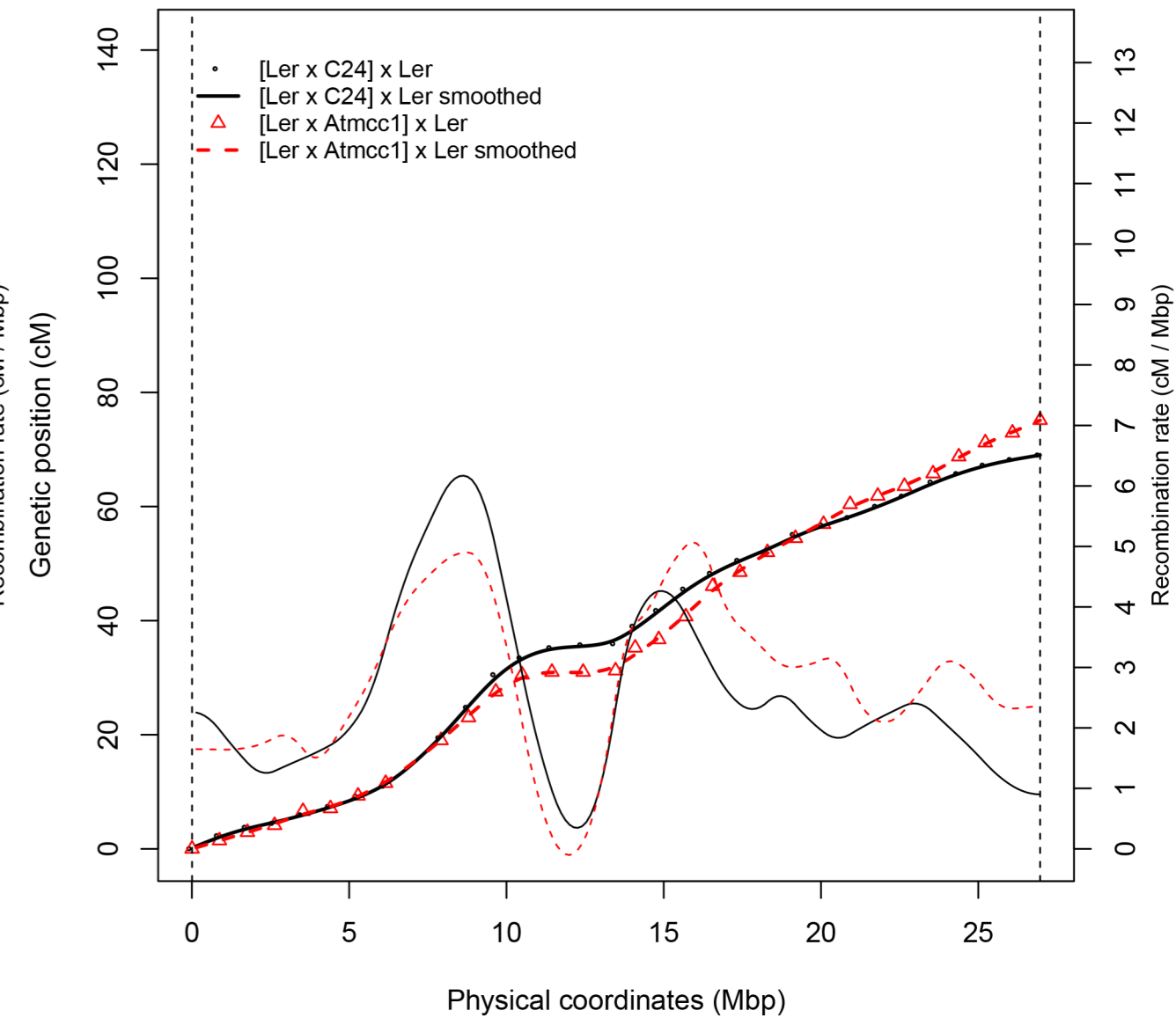

Supplement: Supplementary file 1 — Figure S1. Relationship between physical and genetic positions of the markers on each chromosome, and corresponding recombination rates in the female control and female mutant linkage maps. [file TPJ-100-1163-s001.pdf]

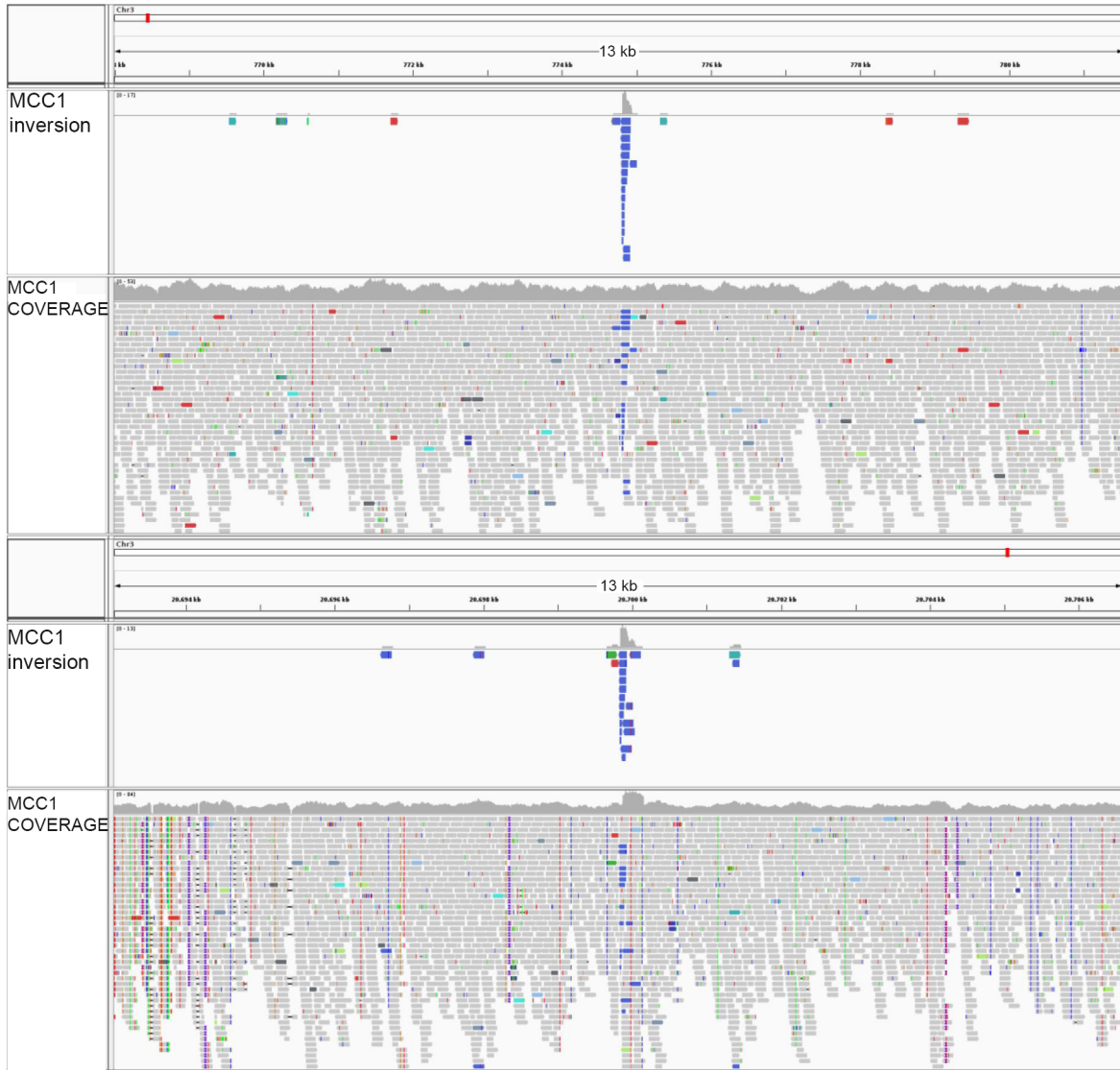

Supplement: Supplementary file 3 — Figure S3. Genome browser visualization of the reads supporting the pericentromeric inversion in Atmcc1. [file TPJ-100-1163-s003.pdf]

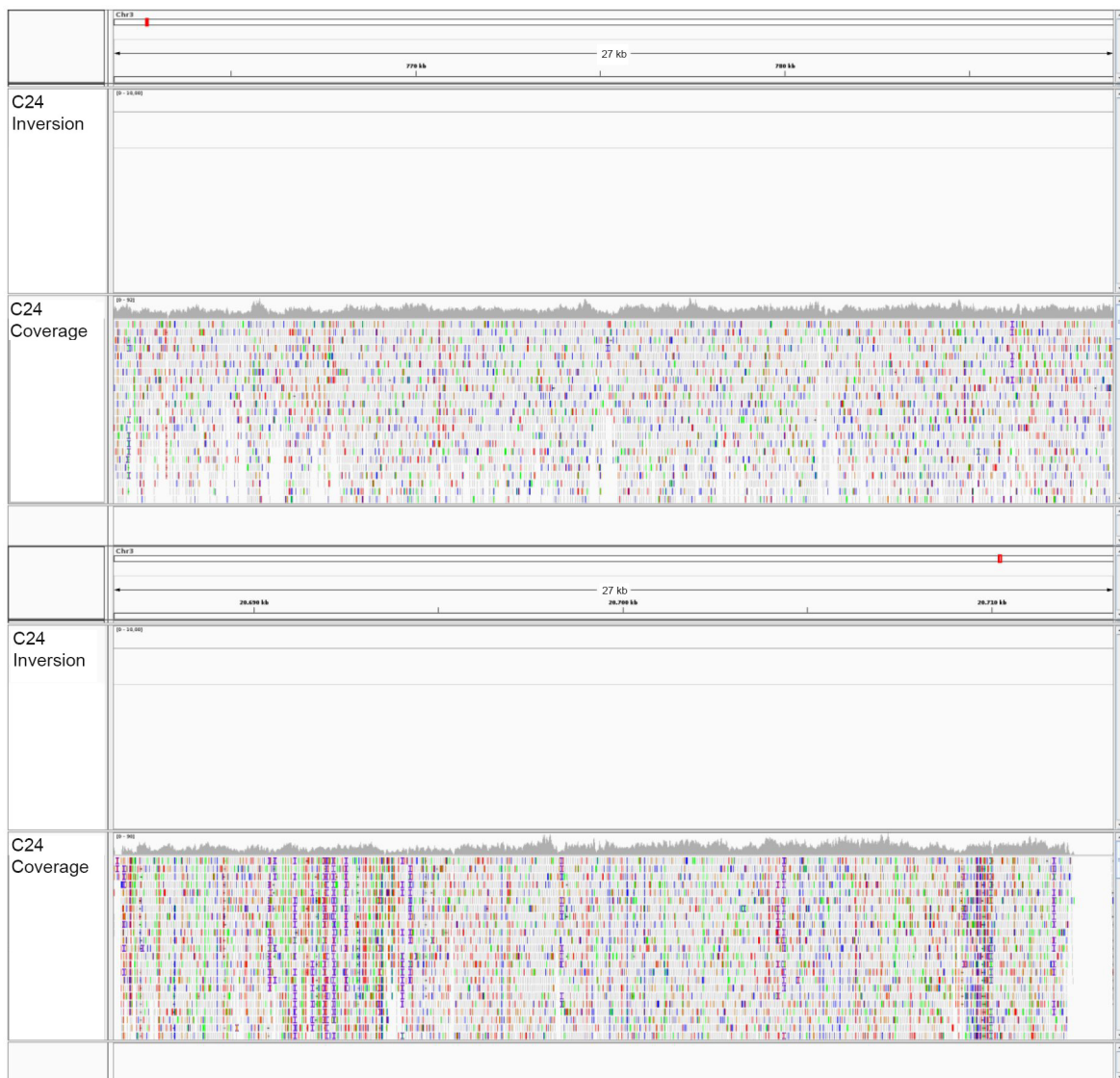

Supplement: Supplementary file 4 — Figure S4. Genome browser visualization of the read coverage in the control C24. [file TPJ-100-1163-s004.pdf]

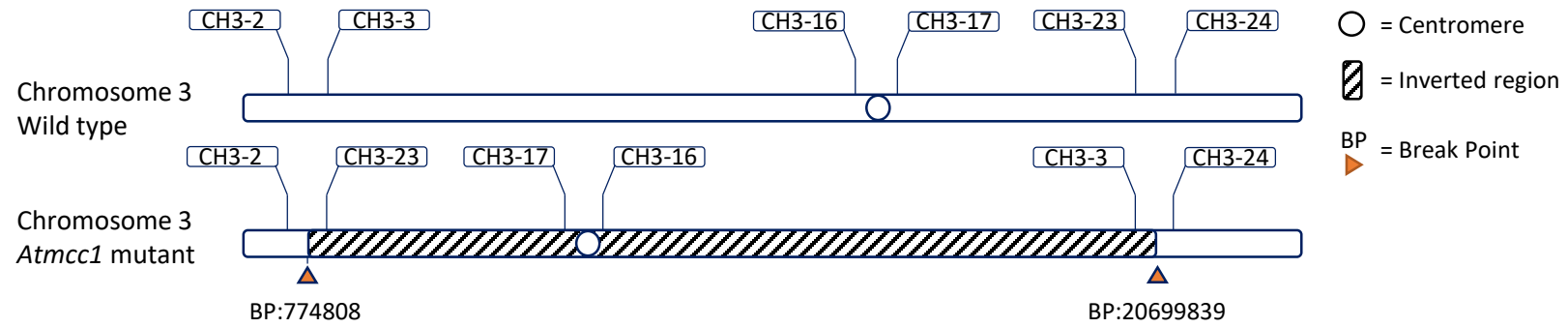

Supplement: Supplementary file 5 — Figure S5. Schematic view of chromosome 3 in control and mutant. [file TPJ-100-1163-s005.pdf]

[Ler x C24] x Ler

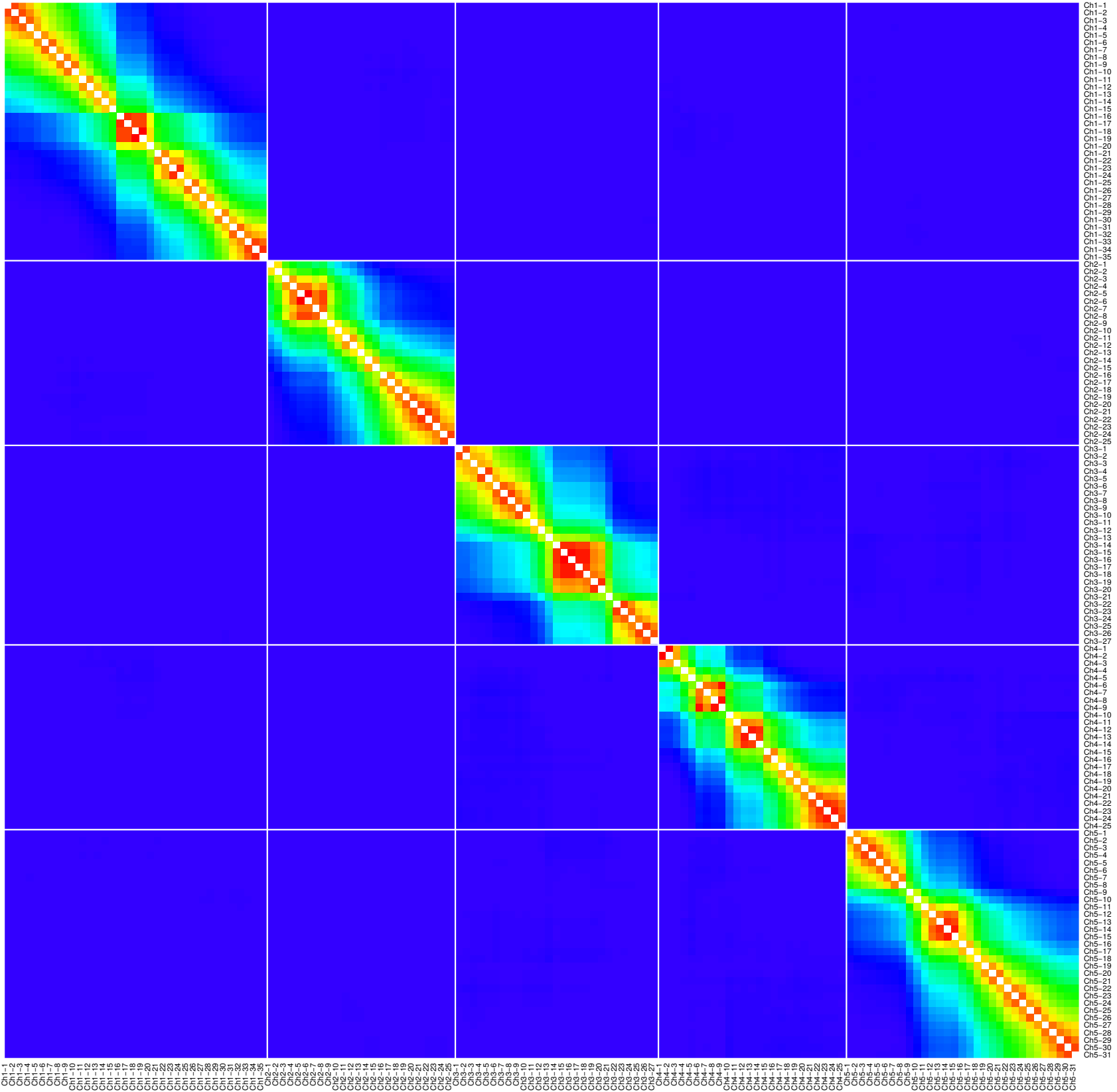

[Ler x Atmcc1] x Ler

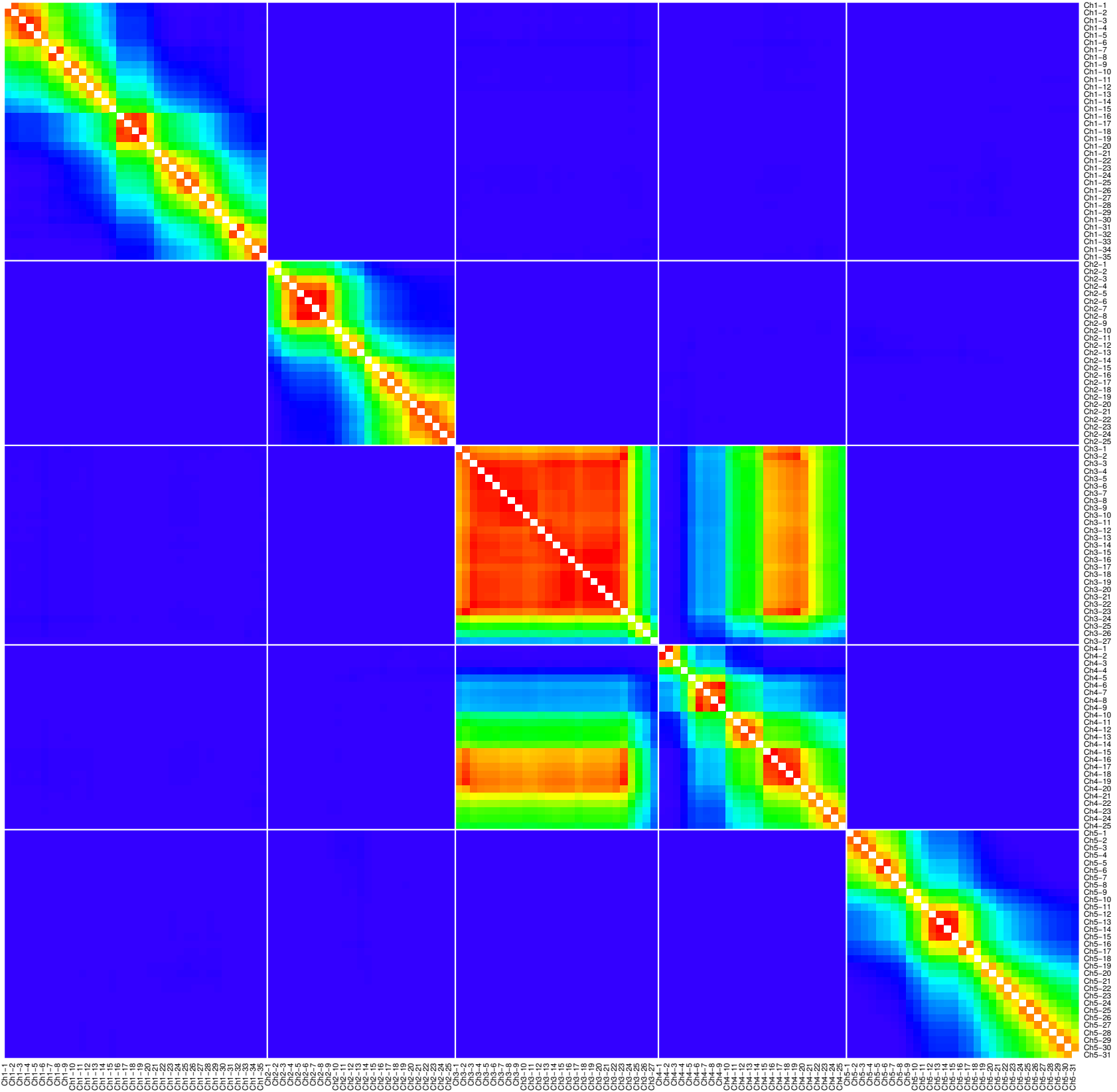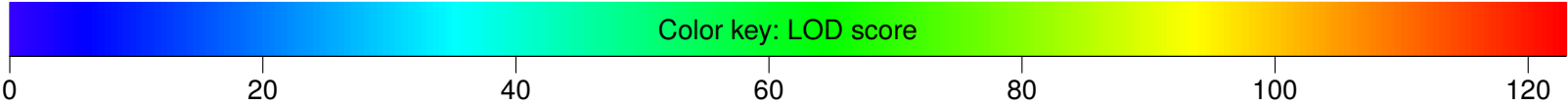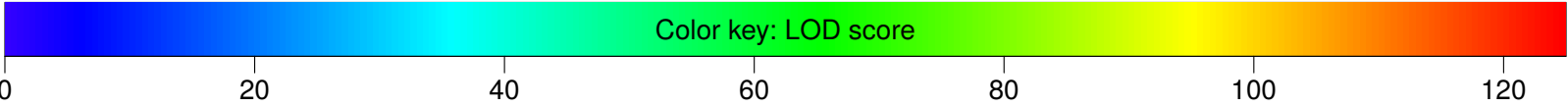

Supplement: Supplementary file 6 — Figure S6. Genome‐wide heat maps of logarithm of the odds scores for pairwise marker linkage in female meiosis. [file TPJ-100-1163-s006.pdf]

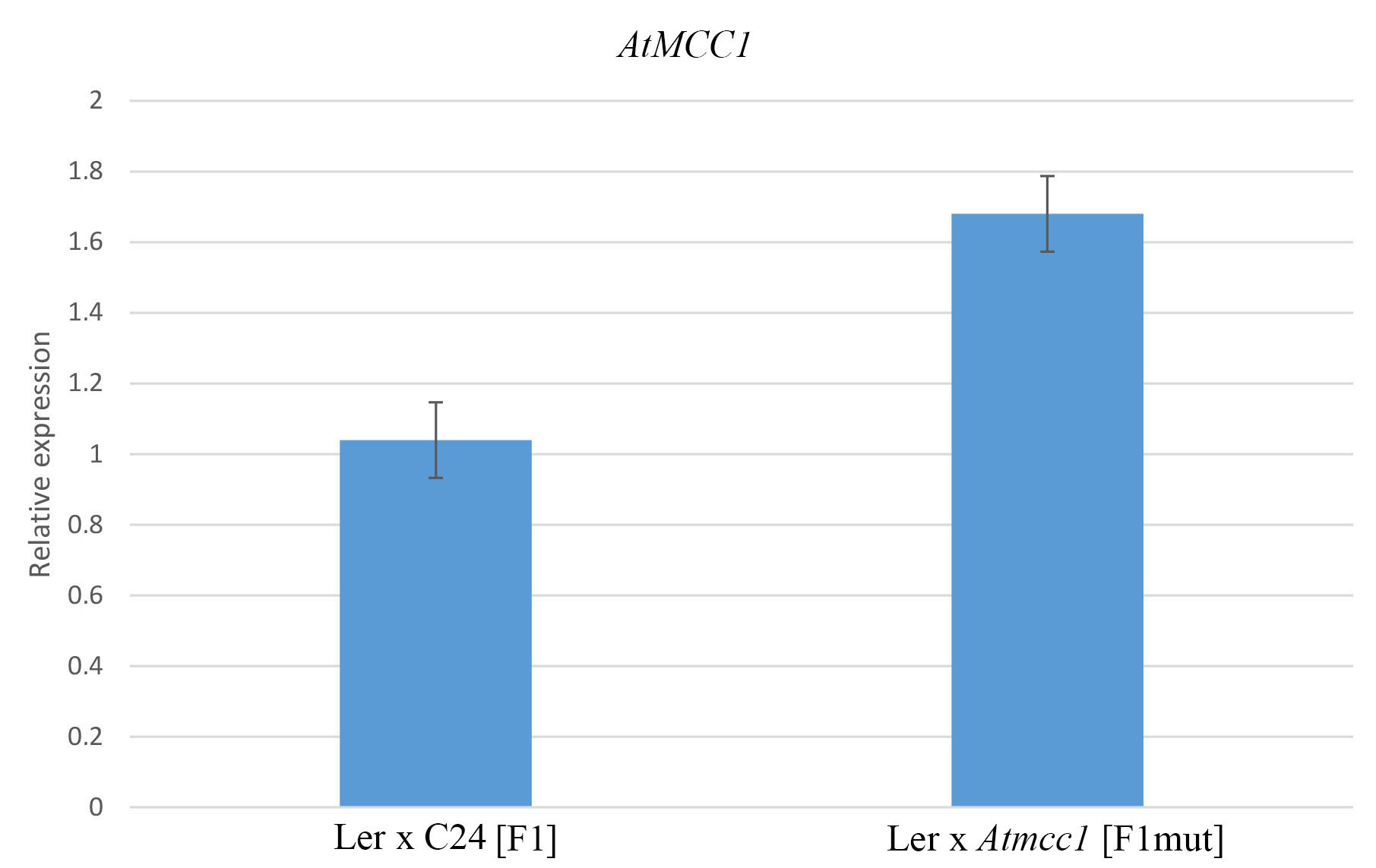

Supplement: Supplementary file 8 — Figure S8. Real‐time RT‐qPCR of AtMCC1 transcript in leaf of Ler × Atmcc1 F1 mutant plants compared with Ler × C24 F1 control plants. [file TPJ-100-1163-s008.jpg]

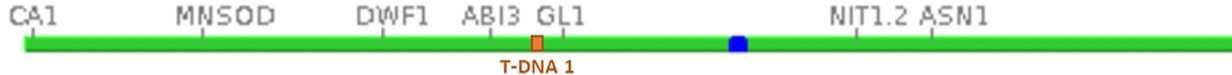

C24

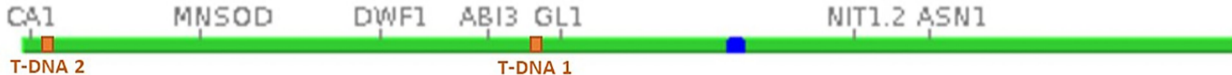

*Atmcc1*

Supplement: Supplementary file 9 — Figure S9. Schematic representation of chromosome 3 indicating some random loci on short and long arm, centromere and T‐DNA insertions in C24 and in Atmcc1. [file TPJ-100-1163-s009.pdf]

4,44

- 
- C:C ●
- C:T ●
- T:T ●
- NTC ●
- ? ●
- Missing ●
- Bad ●
- Short ●
- Dupe ●
- Over ●
- Uncallable ●

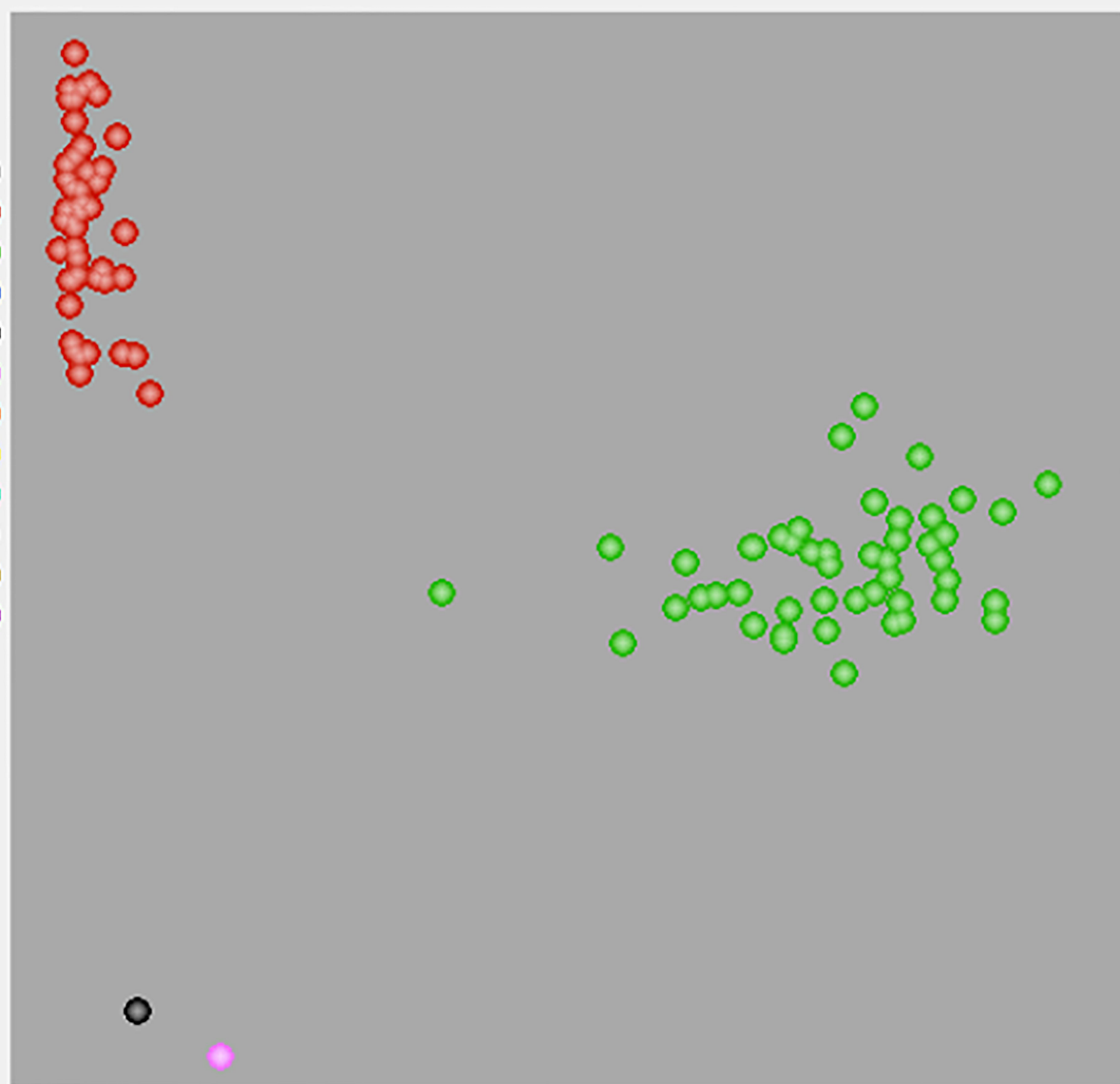

0,15

0,34

3,25

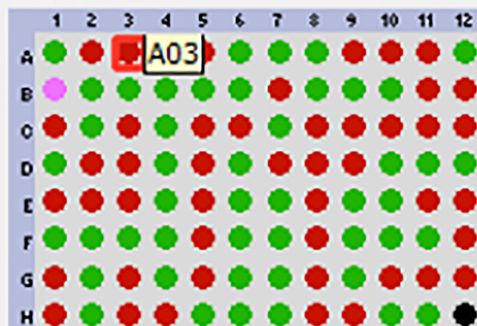

Plate

B1- 00029493

Supplement: Supplementary file 10 — Figure S10. Example of KASP genotyping output. [file TPJ-100-1163-s010.pdf]

# Metr

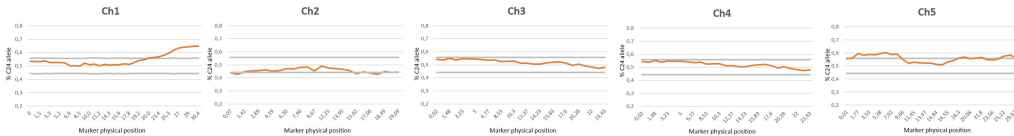

# Mmut

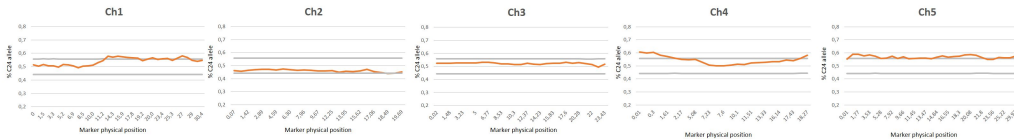

# Fctr

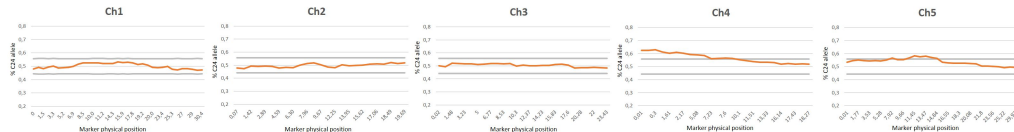

# Fmut

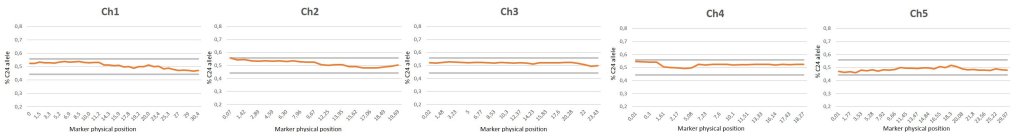

Supplement: Supplementary file 11 — Figure S11. Frequency of C24 allele (in orange) at each marker per chromosome in the mapping of the Mctr population. [file TPJ-100-1163-s011.pdf]
