# Supplementary material for: Recombination suppression in heterozygotes for a pericentric inversion induces the interchromosomal effect on crossovers in Arabidopsis
Source: Plant J. 2019 Oct 7;100(6):1163–75. doi: 10.1111/tpj.14505 (PMC6973161; doi:10.1111/tpj.14505)
Supplement: Supplementary file 2 — Figure S2. Heat maps for the two‐point linkage logarithm of the odds scores on chromosome 3 with the inversion in female meiosis. [file TPJ-100-1163-s002.pdf]

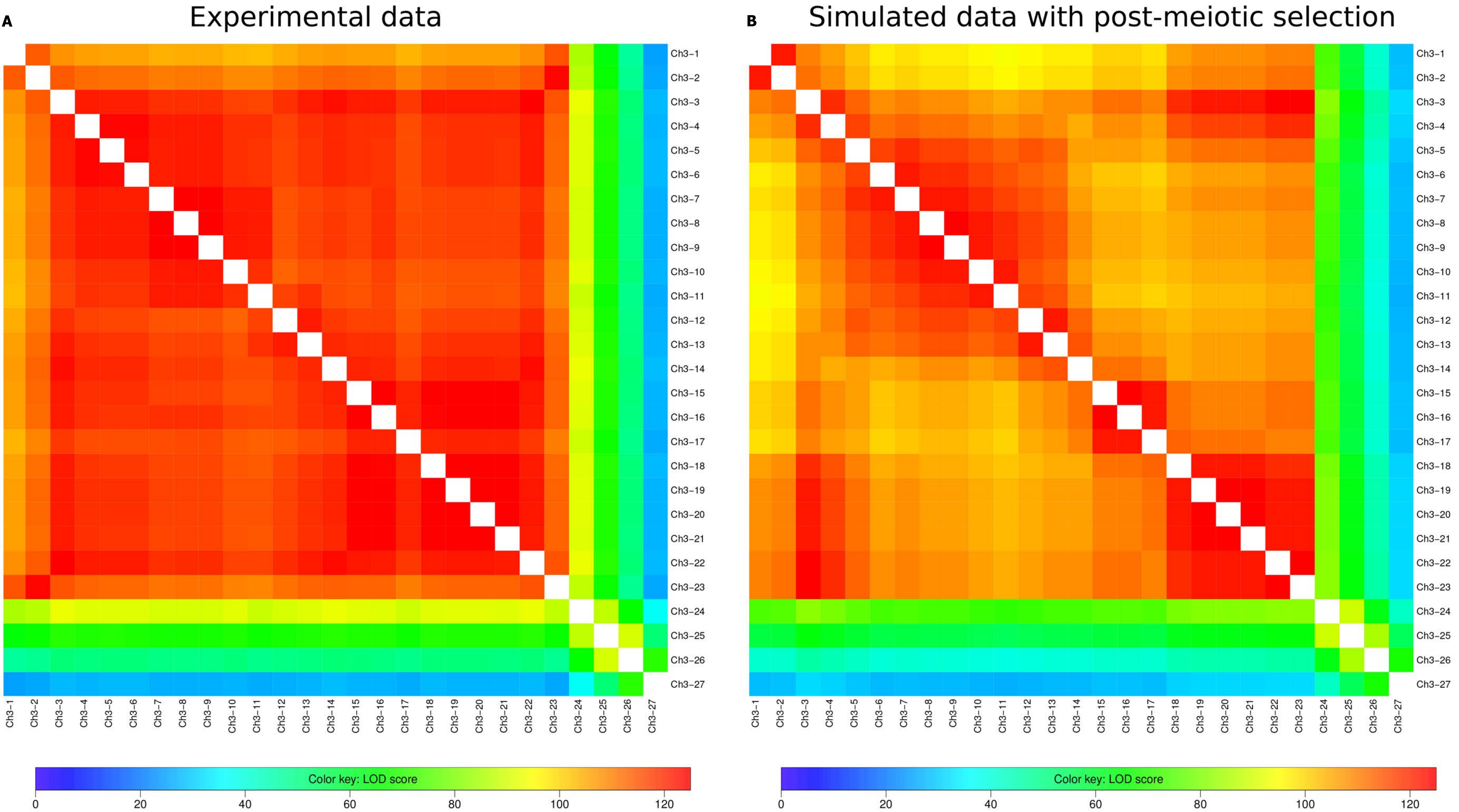

A
Experimental data
B
Simulated data with post-meiotic selection
Ch3−1
Ch3−2
Ch3−3
Ch3−4
Ch3−5
Ch3−6
Ch3−7
Ch3−8
Ch3−9
Ch3−10
Ch3−11
Ch3−12
Ch3−13
Ch3−14
Ch3−15
Ch3−16
Ch3−17
Ch3−18
Ch3−19
Ch3−20
Ch3−21
Ch3−22
Ch3−23
Ch3−24
Ch3−25
Ch3−26
Ch3−27
Color key: LOD score
0
20
40
60
80
100
120
