# Supplementary material for: Recombination suppression in heterozygotes for a pericentric inversion induces the interchromosomal effect on crossovers in Arabidopsis
Source: Plant J. 2019 Oct 7;100(6):1163–75. doi: 10.1111/tpj.14505 (PMC6973161; doi:10.1111/tpj.14505)
Supplement: Supplementary file 7 — Figure S7. Illustration of the statistical test used to compare recombination landscapes between control and mutant populations for chromosomes 1, 2 and 5. [file TPJ-100-1163-s007.pdf]

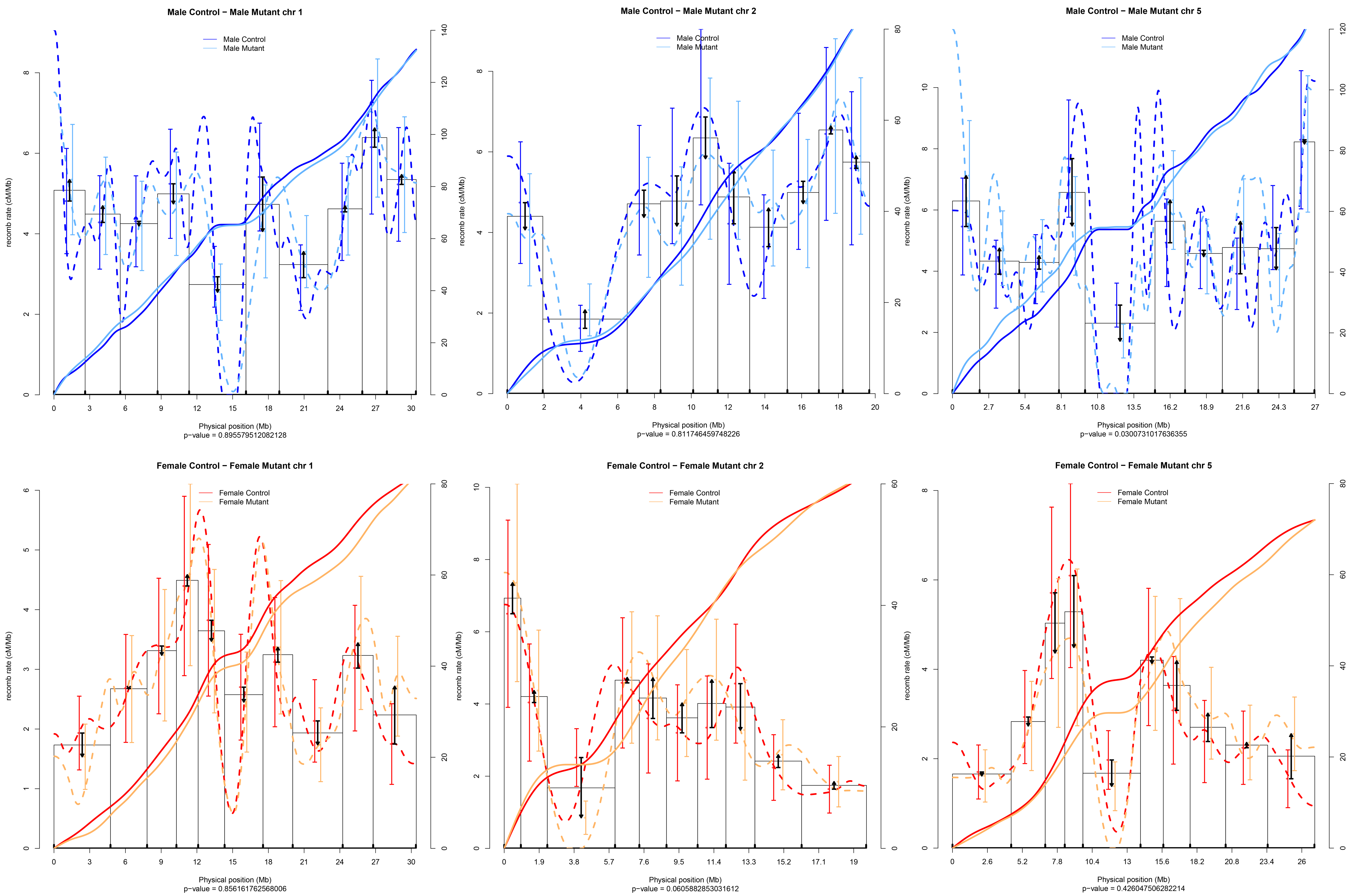
Male Control – Male Mutant chr 1
Male Control
Male Mutant
recomb rate (cM/Mb)
Physical position (Mb)
p-value = 0.895579512082128
Male Control – Male Mutant chr 2
Male Control
Male Mutant
recomb rate (cM/Mb)
Physical position (Mb)
p-value = 0.811746459748226
Male Control – Male Mutant chr 5
Male Control
Male Mutant
recomb rate (cM/Mb)
Physical position (Mb)
p-value = 0.0300731017636355
Female Control – Female Mutant chr 1
Female Control
Female Mutant
recomb rate (cM/Mb)
Physical position (Mb)
p-value = 0.856161762568006
Female Control – Female Mutant chr 2
Female Control
Female Mutant
recomb rate (cM/Mb)
Physical position (Mb)
p-value = 0.0605882853031612
Female Control – Female Mutant chr 5
Female Control
Female Mutant
recomb rate (cM/Mb)
Physical position (Mb)
p-value = 0.426047506282214
