# Supplementary material for: Recombination suppression in heterozygotes for a pericentric inversion induces the interchromosomal effect on crossovers in Arabidopsis
Source: Plant J. 2019 Oct 7;100(6):1163–75. doi: 10.1111/tpj.14505 (PMC6973161; doi:10.1111/tpj.14505)
Supplement: Supplementary file 15 [file TPJ-100-1163-s015.docx]

**FULL SUPPORTING LEGENDS**

**Figure S1. Relationship between physical (Mbp) and genetic positions (cM) of the markers on each chromosome, and corresponding recombination rates (cM/Mbp) in Female Control (black) and Female Mutant (red) linkage maps.** In the bar under each graph, the black box indicates approximate centromere position according to Arabidopsis’ TAIR.

**Figure S2. Heat maps for the two-point linkage LOD scores on chromosome 3 with the inversion in female meiosis.** A) experimental data. B) prediction of the model simulating pairing, crossover formation and post-meiotic selection. Note the characteristic block structure of these heat maps in which the boundaries of the inverted region are tightly linked (high LOD score).

**Figure S3. Genome browser visualization of the reads supporting the pericentromeric inversion in *Atmcc1*.** The top shows the region between the markers Chr3-2 and Chr3-3, centered on the North breakpoint of the inversion (Chr3:774808), while on the bottom the region is centered on the South breakpoint of the inversion (Chr3:20699839) between the markers Chr3-23 and Chr3-24. In both the panels, the first picture shows only the reads supporting the inversion (in blue) whereas the second shows all the reads mapped in the region (whole coverage). Colored lines within the reads indicate mismatches between the sequence of the read and the reference genome.

**Figure S4. Genome browser visualization of the read coverage in the control C24.** The top shows the region between the markers Chr3-2 and Chr3-3, centered on the position where North breakpoint of the inversion was mapped in Atmcc1 (Chr3:774808). On the bottom the region is centered on the position where the South breakpoint of the inversion was found in Atmcc1 (Chr3:20699839) between the markers Chr3-23 and Chr3-24.

**Figure S5. Schematic view of chromosome 3 in control and mutant**. Markers flanking the inversion and the centromere and the exact position of the inversion breakpoints are indicated.

**Figure S6. Genome-wide heat maps of LOD scores for pairwise linkage in female meiosis.** Left: control population showing the standard structure of LOD decreasing as one goes away from the diagonal. No significant LODs appear between markers on different chromosomes. Right: mutant population showing the standard structure for chromosomes 1, 2 and 5 and abnormal behavior both within chromosome 3 and between chromosomes 3 and 4.

**Figure S7. Illustration of the statistical test used to compare recombination landscapes between control and mutant populations for chromosomes 1, 2, and 5.** Solid curves: Marey maps normalized to the average genetic length, so the comparison focuses on differences in the shape of the recombination landscapes and is not affected by differences in the values of chromosome genetic lengths. Dotted curves: derivative of the normalized Marey maps, indicating the recombination landscape along the chromosome. The black rectangles show the ten bins used for the analysis. Bin boundaries were chosen so each bin contained regions of the same genetic length. On top of each bar, a black vertical arrow indicates the difference between both populations in average recombination rates over the bin considered, and the error bars indicate the 95% confidence intervals of these average recombination rates. *p*-values corresponding to the H0 hypothesis that both populations have the same recombination landscape are indicated below the X-axis label.

**Figure S8. Real‐time RT‐qPCR of *AtMCC1* transcript in leaf of Ler x *Atmcc1* F_1_ mutant plants compared to Ler x C24 F_1_ control plants.** T-test was significant (p value <0.05)

**Figure S9. Schematic representation of chromosome 3 indicating some random loci on short and long arm, centromere (blu) and T-DNA insertions (orange) in C24 and in *Atmcc1*.** The first insertion (T-DNA 1) is present in both C24 and *Atmcc1* while the second insertion (T-DNA 2) occurs only in *Atmcc1*.

**Figure** **S10. Example of KASP genotyping output.** Red dots represent Ler homozygous plants, green dots represent Ler/C24 heterozygous plants. Each plate contains a blank control (black dot) and may contain an ambiguous genotype score (pink dots).

**Figure S11. Frequency of C24 allele (in orange) at each marker per chromosome in the mapping of Mctr population.** Lines (grey) represent 99% confidence intervals of the expected 0.5 value under Mendelian segregation.

**Table S1.** **List of SNPs used for genotyping.**

**Table S2. Interference strength measured by fitting the data to a two-pathway Gamma model.** Nu: interference intensity in the interfering pathway (Class I crossovers). p: proportion of crossovers formed via the non-interfering pathway (Class II crossovers). nu_Inf, nu_Sup, p_Inf, p_Sup: lower (Inf) and upper (Sup) boundaries of 95% confidence intervals for nu and p, based on 1000 resimulations.

**Table S3.** **Genotyping scores of the recombinant BC1 populations.** 0 value represents Ler marker at homozygous state while 1 value is the presence of C24 marker.
